# Enhancing acetaminophen solubility and stability using an aqueous betaine chloride–acetic acid natural deep eutectic solvent: experimental and mechanistic insights

**DOI:** 10.1039/d6ra05672e

**Published:** 2026-10-12

**Authors:** The-Huan Tran, Thao Nhu Thi Nguyen, Quynh Phuong Nguyen, Nhan Trong Le, Hoai Thi Nguyen

**Affiliations:** a Faculty of Pharmacy, Hue University of Medicine and Pharmacy, Hue University Hue City Vietnam nthoai@hueuni.edu.vn ltrongnhan@hueuni.edu.vn

## Abstract

Improving the aqueous solubility of moderately soluble drugs using safe and sustainable solvent systems remains an important challenge in pharmaceutical formulation. In this study, a series of aqueous natural deep eutectic solvents (NADESs) were systematically designed and evaluated to enhance the solubility, stability, and biocompatibility of acetaminophen (ACP). The effects of hydrogen bond donor type, hydrogen bond acceptor type, molar ratio, and NADES concentration were investigated to identify the optimal formulation. Among the tested systems, the aqueous betaine chloride–acetic acid NADES (BeCl–Ace) at a molar ratio of 1 : 4 and a concentration of 70% exhibited the highest solubilization capacity, increasing ACP solubility by 25.28 and 22.14-fold at 25 and 37 °C, respectively, compared with water. Computational analyses based on electrostatic potential mapping, binding energy calculations, and interaction region indicator analysis revealed that the enhanced solubility originates from cooperative hydrogen bonding, electrostatic attraction, and van der Waals interactions between ACP and the BeCl–Ace components, particularly BeCl. In addition, the selected NADES formulation significantly improved ACP stability under thermal stress, retaining 89.13% of the initial drug content after 30 days at 37 °C, compared with 70.20% in water. Cytotoxicity evaluation on HEK-293A cells further confirmed good biocompatibility, with cell viability remaining above 92% across the tested concentration range. These findings indicate that aqueous BeCl–Ace NADESs represent a promising and sustainable solvent system for improving ACP formulation performance.

## Abstract 2

Mechanistic insights and experimental evidence reveal an aqueous betaine chloride–acetic acid natural deep eutectic solvent as an effective green platform for enhancing acetaminophen formulation performance.

## Graphical abstract

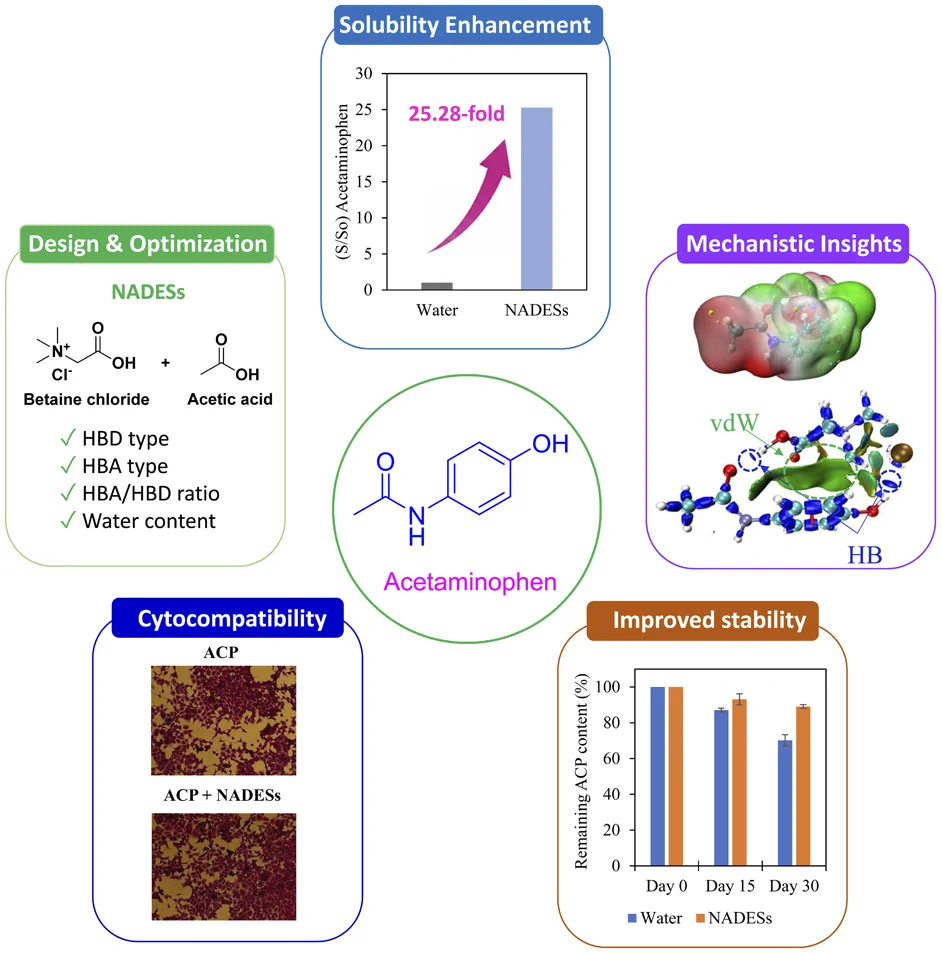

## 1. Introduction

The increasing cost, time, and risk associated with the discovery of new therapeutic agents have intensified interest in formulation strategies that improve the performance of existing drugs.^1,2^ Among these strategies, solubility enhancement is particularly important because inadequate dissolution can limit drug absorption, restrict formulation flexibility, and compromise therapeutic performance.^3–5^ Reformulating marketed active pharmaceutical ingredients using safer and more sustainable solvent systems therefore represents an attractive route to improve drug utility while reducing both economic and environmental burdens.^6^

Acetaminophen (ACP, *N*-(4-hydroxyphenyl)acetamide) is one of the most widely used analgesic and antipyretic drugs because of its established clinical efficacy, good tolerability, and broad use across different patient populations. However, its moderate aqueous solubility, approximately 14 mg mL^−1^ at 25 °C, can restrict the development of concentrated liquid formulations and may limit dissolution performance under certain formulation conditions.^7^ Conventional approaches used to improve ACP solubility include co-solvency, hydrotropic solubilization, surfactants, cyclodextrin complexation, pH adjustment, solid dispersion, and particle size reduction. Although these methods can be effective, many of them rely on synthetic excipients or volatile organic solvents, which may raise concerns regarding residual solvent toxicity, environmental impact, formulation safety, and regulatory acceptability.^7,8^

Natural deep eutectic solvents (NADESs) have emerged as promising green solvent systems for pharmaceutical applications.^6^ They are typically formed by combining a hydrogen bond acceptor (HBA), such as choline chloride or betaine, with a hydrogen bond donor (HBD), such as organic acids, sugars, polyols, amino acids, or amides. The extensive hydrogen bonding network between these components produces liquid eutectic mixtures with melting points lower than those of the individual constituents.^9,10^ Owing to their negligible volatility, tunable polarity, high hydrogen bonding capacity, water compatibility, and potential biocompatibility, NADESs have been increasingly explored as alternative co-solvents for poorly or moderately water soluble drugs.^11^

Several studies have demonstrated that NADESs can enhance ACP solubility in aqueous media.^7,8,12–15^ Most previous investigations mainly focused on choline chloride based NADESs containing urea, oxalic acid, malonic acid, ethylene glycol, glycerol, malic acid, tartaric acid, or 1,2-propanediol, and ACP solubility was generally found to increase with increasing NADES concentration and temperature. These studies provided valuable thermodynamic and physicochemical information, including solubility modeling, activity coefficients, apparent molar volumes, and compressibility parameters. Nevertheless, the current understanding of ACP solubilization in NADESs remains incomplete. Most previous studies have primarily focused on choline chloride-based systems and relied mainly on macroscopic thermodynamic analyses, whereas the potential of betaine chloride based NADESs for ACP solubilization remains largely unexplored. Compared with choline chloride, betaine chloride contains a carboxyl functional group in addition to the quaternary ammonium center, which may provide a distinct interaction environment and stronger intermolecular interactions with ACP molecules. In addition, direct molecular level evidence regarding the dominant interaction sites and the relative contributions of hydrogen bonding, electrostatic attraction, and dispersion interactions is still limited.

Furthermore, previous studies have mainly emphasized solubility enhancement and thermodynamic correlation, while the storage stability and cytotoxicity of ACP loaded aqueous NADES formulations have rarely been evaluated in an integrated manner. Such evaluations are particularly important because a solvent system with high solubilization capacity may not necessarily be suitable for pharmaceutical applications if it compromises drug stability or induces cellular toxicity. Moreover, the rational selection of HBA, HBD, molar ratio, and water content requires mechanistic insight into how each component contributes to the stabilization of ACP in the dissolved state. Therefore, a more comprehensive formulation framework integrating solubility enhancement, molecular interaction analysis, storage stability, and preliminary biocompatibility evaluation is still required for the rational development of NADES based pharmaceutical solvent systems.

In this study, a series of aqueous NADESs were rationally designed and systematically screened to enhance the solubility of ACP. The effects of HBD type, HBA type, HBA/HBD molar ratio, and NADES concentration were investigated to identify the optimal formulation. The stability of ACP in the selected NADES formulation was compared with that in water under different storage temperatures, while cytotoxicity assays using HEK-293A normal human cells were conducted to assess preliminary biocompatibility. Furthermore, electrostatic potential (ESP) and interaction region indicator (IRI) analyses were applied to elucidate the molecular interactions responsible for ACP solubilization. Distinct from previous studies that have predominantly focused on individual choline- or betaine-based NADES systems and solubility-related properties, the present study systematically screens different aqueous NADES formulations for ACP, leading to the identification of BeCl–Ace as the optimal system. By integrating systematic formulation screening with molecular-level interaction analysis, stability assessment, and preliminary cytotoxicity evaluation, this study provides both mechanistic insight into ACP solubilization and a basis for further evaluating aqueous NADES for pharmaceutical formulation.

## 2. Materials and methods

### 2.1. Standards, reagents, and solvents

Acetaminophen (ACP) was obtained from Sigma-Aldrich (USA). Choline chloride (ChCl) and betaine chloride (BeCl), proline (Pro), ethylene glycol (Eth), propylene glycol (Prg), acetic acid (Ace), oxalic acid (Oxa), lactic acid (Lac), tartaric acid (Tar), citric acid (Cit), glucose (Glu), fructose (Fru), sucrose (Suc), maltose (Mal), xylitol (Xyl), urea (Ure) were supplied by Xilong Scientific (China) and Macklin (China). All chemicals were of analytical grade and used without further purification. Ultrapure water was used throughout the experiments. For cytotoxicity assays, Dulbecco's Modified Eagle Medium (DMEM), fetal bovine serum (FBS), trypsin–EDTA, and other cell culture reagents were purchased from standard commercial suppliers. The human embryonic kidney cell line (HEK-293A) was kindly provided by the University of Milan (Italy).

### 2.2. Preparation of NADESs

NADESs were prepared by mixing HBA with HBD at predetermined molar ratios (Table 1). The mixtures were heated under continuous stirring at approximately 80 °C until a homogeneous and transparent liquid was obtained. After complete dissolution, the NADESs were allowed to cool to room temperature and stored in sealed containers in a desiccator to prevent moisture absorption. Aqueous NADES formulations were subsequently prepared gravimetrically by mixing the neat NADES with ultrapure water at the required mass fractions. For concentration optimization of the selected BeCl–Ace (1 : 4) system, NADES concentrations ranging from 10 to 100% (w/w) were evaluated. Formulations containing 10–90% (w/w) NADES were prepared by adding the corresponding amount of ultrapure water, whereas the 100% formulation consisted of neat BeCl–Ace (1 : 4) without added water.

**Table 1:** Composition, molar ratio, and concentration of the prepared aqueous NADES formulations used in this study

Solvents	Abbreviation	Molar ratio (mol : mol)	Concentration (%, g g^−1^)
Choline chloride–ethylene glycol	ChCl–Eth	1 : 2	50
Choline chloride–glycerol	ChCl–Gly	1 : 2	50
Choline chloride–propylene glycol	ChCl–Prg	1 : 2	50
Choline chloride–acetic acid	ChCl–Ace	1 : 2	50
Choline chloride–oxalic acid	ChCl–Oxa	1 : 2	50
Choline chloride–lactic acid	ChCl–Lac	1 : 2	50
Choline chloride–tartaric acid	ChCl–Tar	1 : 2	50
Choline chloride–citric acid	ChCl–Cit	1 : 2	50
Choline chloride–glucose	ChCl–Glu	1 : 2	50
Choline chloride–fructose	ChCl–Fru	1 : 2	50
Choline chloride–sucrose	ChCl–Suc	1 : 2	50
Choline chloride–maltose	ChCl–Mal	1 : 2	50
Choline chloride–xylitol	ChCl–Xyl	1 : 2	50
Choline chloride–urea	ChCl–Ure	1 : 2	50
Betaine chloride–acetic acid	BeCl–Ace	1 : 1, 1 : 2, 1 : 3, 1 : 4, 1 : 5	10, 20, 30, 40, 50, 60, 70, 80, 90, 100
Betaine chloride–lactic acid	BeCl–Lac	1 : 2	50
Proline–acetic acid	Pro–Ace	1 : 2	50
Proline–lactic acid	Pro–Lac	1 : 2	50

### 2.3. Determination of ACP solubility

The solubility of ACP in aqueous NADESs was determined using a saturation shake-flask method with slight modifications. An excess amount of ACP was added to each aqueous NADES solution or pure water, followed by incubation at 25 °C and 37 °C under constant agitation at 750 rpm using a ThermoMixer C (Eppendorf, Germany). After 72 h, the samples were centrifuged twice at 10 000 rpm to remove undissolved solids, and the supernatants were collected and filtered through a 0.22 µm nylon membrane filter.^16^ The filtrates were appropriately diluted and analyzed using a UV-Vis spectrophotometer (V-730, Jasco, Japan). Prior to analysis, an ACP calibration curve was established, and sample absorbance was measured at 248 nm, corresponding to the maximum absorption wavelength of ACP.^7^

### 2.4. Computational methods

The molecular structures of ACP, BeCl, Ace, water (H_2_O), and the corresponding 1 : 1 complexes were built in Avogadro^17^ and initially pre-optimized using the MMFF94 force field. The optimized structures were then subjected to quantum chemical calculations with ORCA 6.1.0.^18,19^ Full geometry optimizations were performed at the B3LYP-D3(BJ)/def2-SVP level^20,21^ using the RIJCOSX approximation^22,23^ and the TightSCF convergence criterion.

ESP surfaces were generated to examine the charge distribution of ACP, the solvent components, and their complexes, with the aim of identifying electron-rich and electron-deficient regions involved in favorable intermolecular interactions. The binding energy of each complex was calculated as:Δ*E*_binding_ = *E*_complex_ − (*E*_ACP_ + *E*_solvent_)where *E*_complex_, *E*_ACP_, and *E*_solvent_ are the electronic energies of the optimized complex, ACP, and the corresponding solvent molecule, respectively.

To further characterize the noncovalent interactions between ACP and the solvent molecules, IRI^24^ analysis was carried out. Scatter plots of sign(*λ*_2_)*ρ versus* IRI and the corresponding IRI isosurfaces were used to distinguish hydrogen-bonding regions, van der Waals interactions, and steric repulsion. ESP and IRI analyses were performed with Multiwfn 3.8 (ref. 25 and 26) and visualized using VMD 1.9.4.^27^

### 2.5. Stability study of ACP in aqueous NADES solutions

The stability of ACP in aqueous NADES solutions was evaluated by dissolving ACP in the prepared solutions and pure water at concentrations below their respective solubility limits, followed by recording the initial concentrations. The samples were then stored at 25 °C and 37 °C under controlled relative humidity (75–80%) and protected from light. The temperature of 37 °C was selected as an elevated storage condition to preliminarily evaluate the thermal stability of ACP in the developed NADES formulation under physiologically and pharmaceutically relevant conditions. After storage periods of 15 and 30 days, aliquots were collected and analyzed to determine the remaining concentration of ACP.^28^ The remaining ACP content was determined by UV-Vis spectrophotometry at 248 nm using the ACP calibration procedure described above and was expressed relative to the corresponding initial value. All experiments were performed independently at least twice, and each measurement was conducted in triplicate.

### 2.6. Cytotoxicity assay on normal human cell line

The cytotoxicity of NADESs, ACP, and ACP-loaded NADES formulations was evaluated using the sulforhodamine B (SRB) assay on HEK-293A cells.^29^ Cells were cultured in DMEM supplemented with 10% FBS, 2 mM l-glutamine, and 1.5 g L^−1^ sodium bicarbonate at 37 °C in a humidified atmosphere containing 5% CO_2_. The cells were seeded into 96-well plates at a density of approximately 5700 cells per well and allowed to attach overnight. Test samples at various concentrations were added to each well, followed by incubation for 72 h. After incubation, the cells were fixed with cold 20% (w/v) trichloroacetic acid at 4 °C for 1 h, stained with SRB solution for 30 min at room temperature, and washed with 1% acetic acid to remove excess dye. The bound dye was subsequently solubilized using 10 mM Tris base, and the absorbance was measured at 515 nm using a microplate reader. In addition, cell morphology was observed and recorded using an inverted microscope (Euromex Oxion Inverso, Netherlands). Ellipticine and 0.05% DMSO were used as positive and negative controls, respectively, and all experiments were performed in triplicate.

### 2.7. FTIR and ^1^H NMR characterization

The BeCl–Ace (1 : 4) NADES and its individual components (BeCl and Ace) were characterized by Fourier-transform infrared (FTIR) and proton nuclear magnetic resonance (^1^H NMR) spectroscopy. FTIR spectra were recorded using a Cary 630 FTIR spectrometer (Agilent Technologies, USA) over the range of 4000–650 cm^−1^ at a resolution of 4 cm^−1^, with 8 scans collected for each sample. ^1^H NMR spectra were recorded on an AVANCE III HD 500 MHz spectrometer (Bruker, Germany) using DMSO-d_6_ as the solvent.

### 2.8. Statistical analysis

All experiments were performed using three independent experimental replicates (*n* = 3), with separate samples prepared for each replicate. Data are presented as mean ± standard deviation (SD). Statistical comparisons were performed separately for each screening dataset and at each investigated temperature using one-way analysis of variance (ANOVA) in SPSS v26 (IBM, USA), followed by Fisher's LSD post hoc test when the overall ANOVA was significant. Differences were considered statistically significant at *p* < 0.05.

## 3. Results and discussion

### 3.1. Design of NADES formulations for enhancing ACP solubility

A series of NADESs were rationally designed and systematically evaluated to enhance the solubility of ACP. The effects of HBD, HBA, molar ratio, and water content were investigated to identify the optimal formulation. The solubility of ACP in aqueous NADES solutions was compared with that in pure water under identical conditions.

#### 3.1.1. Effect of HBD on ACP solubility

The influence of HBD type on ACP solubility was initially investigated using a series of choline chloride based NADESs at a fixed molar ratio of 1 : 2 and a fixed concentration. As shown in Fig. 1, all tested NADESs enhanced the solubility of ACP compared with pure water, with solubility enhancement factors ranging from 2.85 to 8.06 at 25 °C and from 1.88 to 7.06 at 37 °C. These results clearly indicate that the chemical nature of the HBD plays a decisive role in governing the solubilization capacity of the NADESs.

**Fig. 1:**
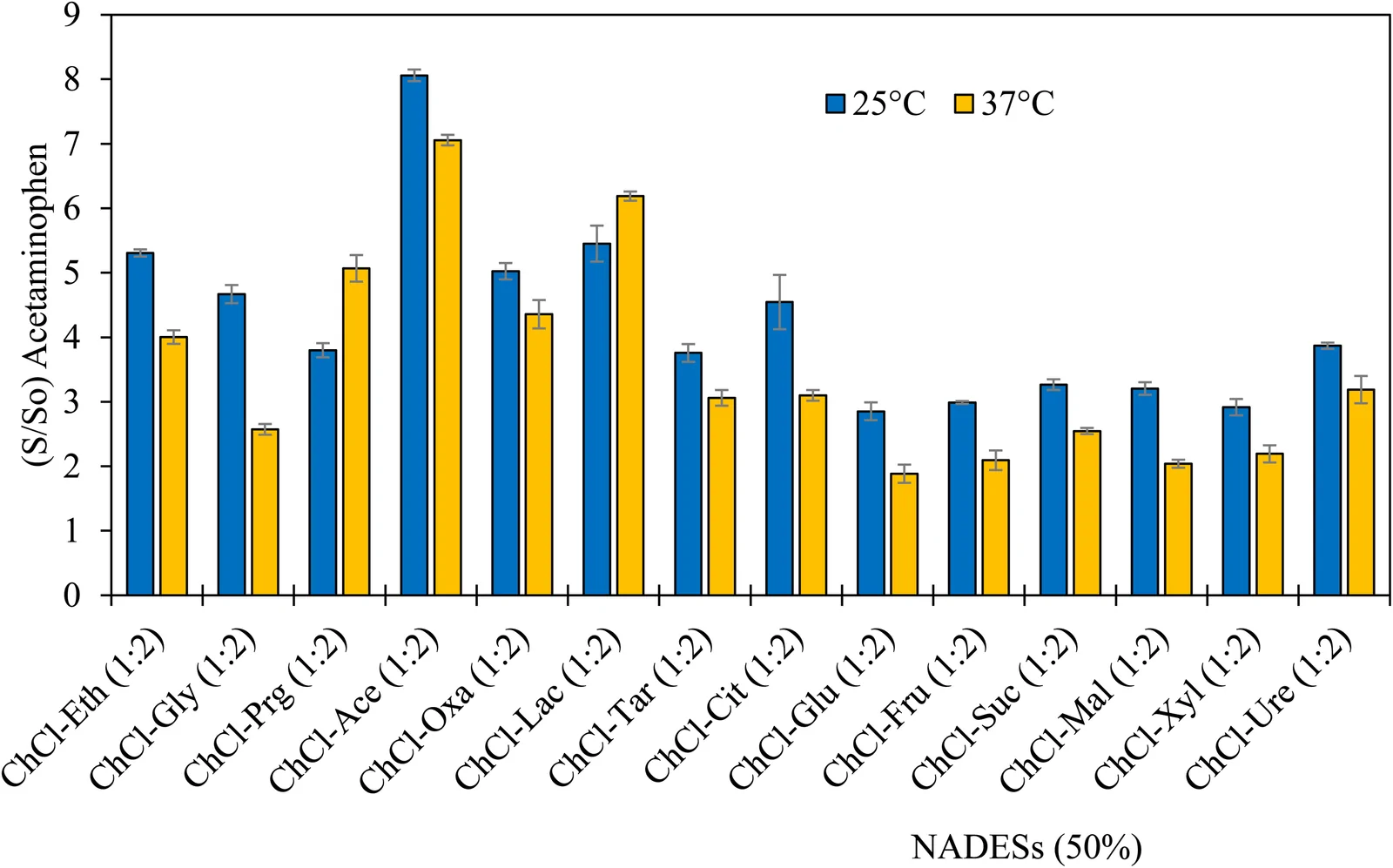
Effect of HBD type on the solubility of ACP in aqueous NADESs (50%) at 25 °C and 37 °C. *S*/*S*_0_ represents the solubility enhancement factor relative to water. ChCl–Ace (1 : 2) achieved the highest *S*/*S*_0_ value, which was significantly greater than those of the other tested NADESs (*p* < 0.05).

A distinct dependence on HBD class was observed. Among the investigated HBD, acid-based NADESs generally exhibited superior solubilization performance relative to alcohol-based and sugar-based NADESs. In particular, ChCl–Ace and ChCl–Lac showed the highest enhancement factors, reaching 8.06 and 5.45 at 25 °C, and 7.06 and 6.19 at 37 °C, respectively. In contrast, sugar-based NADESs such as ChCl–Glu, ChCl–Fru, ChCl–Suc, ChCl–Mal, and ChCl–Xyl gave the lowest enhancement factors, mostly in the range of 2.85 to 3.27 at 25 °C and 1.88 to 2.55 at 37 °C. Alcohol-based NADESs displayed intermediate behavior, with ChCl–Eth and ChCl–Gly showing relatively good performance, whereas ChCl–Prg was more effective at 37 °C than at 25 °C.

The superior performance of acid-based NADESs can be attributed primarily to their strong hydrogen bond donating ability and favorable polarity.^30^ ACP contains both a phenolic hydroxyl group and an amide moiety, allowing it to participate in multiple intermolecular hydrogen bonding interactions.^31^ Therefore, HBD bearing carboxylic acid groups can interact effectively with ACP through hydrogen bonding with the carbonyl oxygen and hydroxyl group of the drug. In addition, the polar environment generated by acid-based NADESs is likely more compatible with the moderately polar structure of ACP, thereby facilitating its transfer from the crystal lattice into the solvent phase.

Besides hydrogen bonding, van der Waals interactions and hydrophobic effects may also contribute to the enhanced solubilization.^32^ ACP contains an aromatic ring that can participate in dispersion interactions with the organic framework of the NADES components. For smaller organic acids such as acetic acid and lactic acid, the relatively compact molecular structure may allow closer molecular packing around ACP, leading to more efficient stabilization of the dissolved state. This effect may partly explain why ChCl–Ace and ChCl–Lac outperformed the other acid based systems.

The effect of carbon chain length also appears to be temperature dependent. Within the alcohol group, the solubilization efficiency followed the order ethylene glycol > glycerol > propylene glycol at 25 °C, whereas at 37 °C propylene glycol became more favorable. This behavior suggests that the influence of carbon chain length is not linear, but rather arises from a balance among polarity, steric effects, hydrogen bonding density, viscosity, and temperature dependent molecular mobility. A slight increase in carbon chain length may enhance hydrophobic interactions with the aromatic moiety of ACP, while elevated temperature can reduce viscosity and facilitate molecular diffusion, thereby modifying the relative contribution of these interactions to solubilization. However, excessive structural bulk or reduced polarity may still weaken the overall solvation efficiency. Similarly, among the acid based NADESs, the best performance was observed for the relatively small acids, acetic acid and lactic acid, whereas acids with larger or more highly functionalized structures such as tartaric acid and citric acid were less effective. This finding suggests that increasing molecular size or the number of functional groups does not necessarily improve solubilization, because overly complex HBD structures may strengthen the internal hydrogen bonding network of the NADES itself and limit the availability of interaction sites for ACP.

The poor performance of sugar-based NADESs can be rationalized by their inherently high viscosity and highly self associated hydrogen bond networks.^33^ Sugars possess multiple hydroxyl groups, which can generate dense intermolecular interactions within the solvent structure. Although these groups provide abundant hydrogen bond sites, they may also promote strong NADES self organization and restrict molecular mobility, thus hindering mass transfer and limiting the ability of the solvent to effectively solvate ACP. In other words, the presence of many hydroxyl groups alone is insufficient to guarantee high solubilization efficiency if the resulting medium becomes too viscous and structurally rigid.

Viscosity is therefore another key factor controlling ACP solubility in NADESs.^6^ In general, NADESs with lower to moderate viscosity are expected to facilitate better diffusion of solvent molecules toward the drug surface and improve disruption of the crystal lattice, whereas highly viscous systems tend to suppress mass transfer and reduce the apparent solubilization rate. This consideration helps explain why acid based NADESs, especially those formed with smaller organic acids, performed better than sugar-based systems. Their lower viscosity and more flexible solvent structure likely promoted more efficient interaction with ACP molecules.

Temperature also influenced the observed trends. For most systems, the enhancement factor at 37 °C was slightly lower than that at 25 °C. This does not necessarily indicate lower absolute solubility in the NADES phase, but rather reflects that the reference solubility of ACP in water also increases with temperature, thereby reducing the *S*/*S*_0_ ratio. Nevertheless, the consistently high values obtained for ChCl–Ace and ChCl–Lac at both temperatures confirm the robustness of these systems as promising candidates for ACP solubilization.

Overall, the results indicate that effective HBD for ACP solubilization should possess an appropriate balance of hydrogen bond donating ability, moderate molecular size, suitable polarity, and manageable viscosity. Among the investigated HBD, acetic acid and lactic acid best fulfilled these requirements, and were therefore selected for subsequent screening of HBA type and further optimization.

#### 3.1.2. Effect of HBA on ACP solubility

Based on the results of the initial screening, Lac and Ace were identified as the most effective HBD for enhancing ACP solubility, with ChCl–Ace exhibiting the highest solubilization efficiency, followed by ChCl–Lac. However, the effect of HBA may alter the relative solubilization performance of NADESs based on different HBD. Therefore, the influence of HBA type was further investigated using these selected HBD in combination with different HBA, including ChCl, BeCl, and Pro. The results are presented in Fig. 2.

**Fig. 2:**
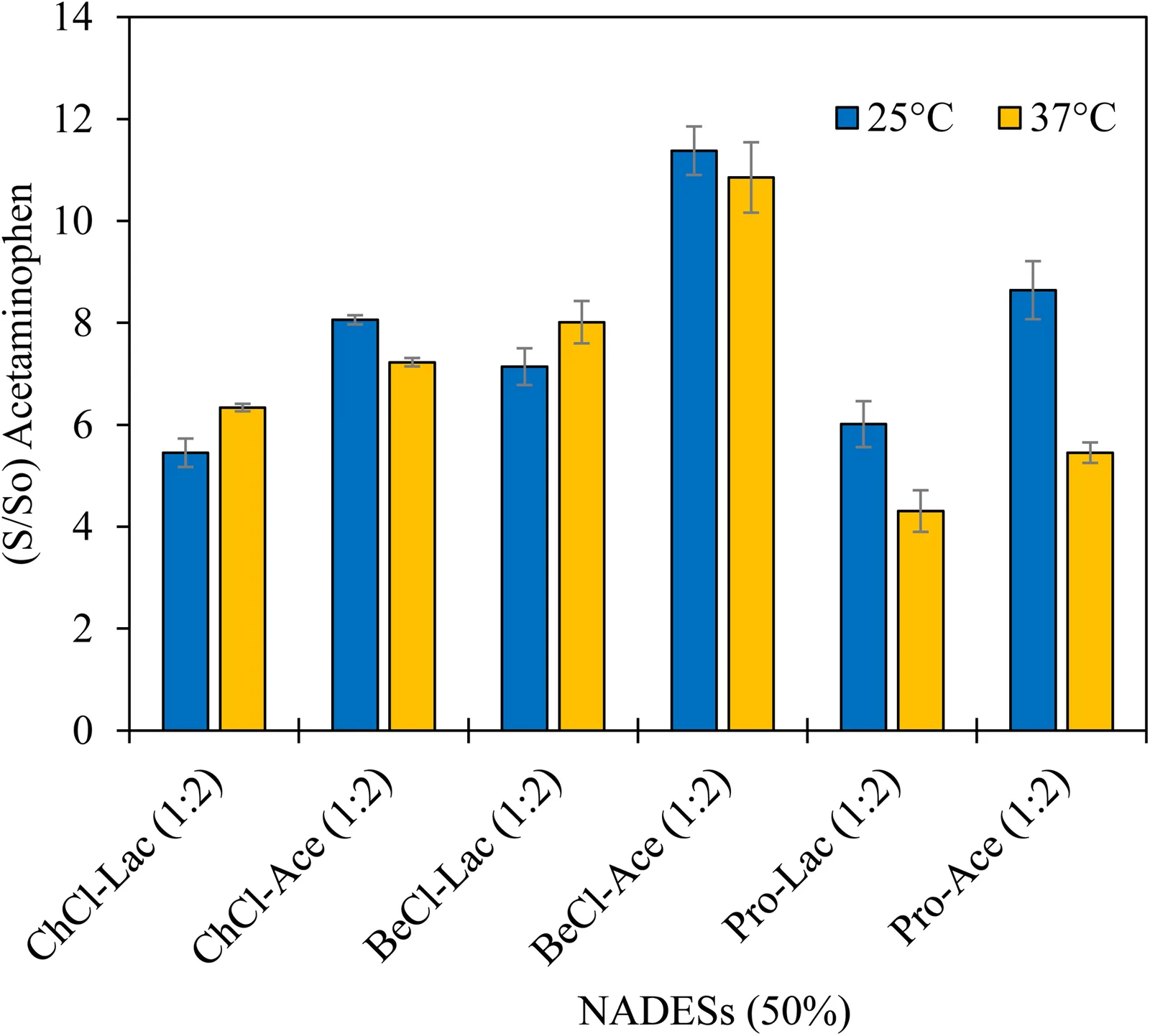
Effect of HBA on the solubility of ACP in aqueous NADESs (50%) at 25 °C and 37 °C using lactic acid and acetic acid as HBD. *S*/*S*_0_ represents the solubility enhancement factor relative to water. BeCl–Ace (1 : 2) achieved the highest *S*/*S*_0_ value, which was significantly greater than those of the other tested NADESs (*p* < 0.05).

As shown in Fig. 2, the types of HBA had a pronounced effect on the solubility of ACP. A consistent trend was observed across all investigated systems, in which NADESs based on acetic acid generally exhibited higher solubility enhancement compared to those based on lactic acid, indicating that acetic acid provides a more favorable solvation environment for ACP under identical HBA conditions. Among the investigated HBA, BeCl-based NADESs exhibited the highest solubilization efficiency, followed by those based on ChCl and Pro. In particular, BeCl–Ace (1 : 2) showed the highest enhancement factor, reaching 11.38 at 25 °C and 10.85 at 37 °C, while BeCl–Lac (1 : 2) also demonstrated superior performance with values of 7.14 and 8.01 at the respective temperatures. In contrast, Pro-based NADESs showed relatively lower solubilization capacity, especially at 37 °C, where a significant decrease was observed.

The superior performance of BeCl-based NADESs can be attributed to the distinct structural characteristics of BeCl, particularly the presence of a carboxyl functional group together with a quaternary ammonium moiety within the same molecular framework. This combination provides multiple interaction sites that can promote stronger electrostatic attraction and more versatile hydrogen bonding interactions with ACP molecules. In particular, the carboxyl group in BeCl can act as both a hydrogen bond donor and acceptor, enabling more effective interaction with the phenolic hydroxyl and amide functionalities of ACP, thereby enhancing molecular stabilization in the solvent environment and promoting higher solubility. In comparison, ChCl-based NADESs also exhibited good solubilization performance owing to the presence of the quaternary ammonium center and hydroxyl group, which contribute to hydrogen bonding and polar interactions. However, replacing the hydroxyl functionality in ChCl with the carboxyl group in BeCl appears to provide a more favorable interaction environment for ACP, highlighting the important role of the carboxyl group in strengthening solute–solvent interactions and improving solubilization efficiency. On the other hand, Pro-based NADESs showed inferior performance, which may be associated with the bulkier cyclic structure and lower charge accessibility of proline. The steric constraints imposed by the pyrrolidine ring may limit effective molecular packing and reduce the flexibility of intermolecular interactions with ACP, resulting in a less favorable hydrogen bonding network for efficient solubilization.

Furthermore, the observed trends were consistent across both HBD, indicating that the effect of HBA is independent but synergistic with the selected HBD. Notably, the combination of BeCl with acetic acid resulted in the highest solubility enhancement, suggesting a strong synergistic effect between these two components. Overall, these results demonstrate that the choice of HBA significantly influences the solubilization behavior of ACP in NADESs. Among the tested HBA, betaine chloride was identified as the most effective component, and therefore BeCl–Ace was selected as the optimal formulation for subsequent experiments.

#### 3.1.3. Effect of HBA/HBD molar ratio on ACP solubility

Following the identification of BeCl–Ace as the optimal NADES formulation, the effect of HBA/HBD molar ratio on ACP solubility was systematically investigated. The molar ratio between betaine chloride and acetic acid was varied from 1 : 1 to 1 : 5 while maintaining a constant NADES concentration. The results are presented in Fig. 3.

**Fig. 3:**
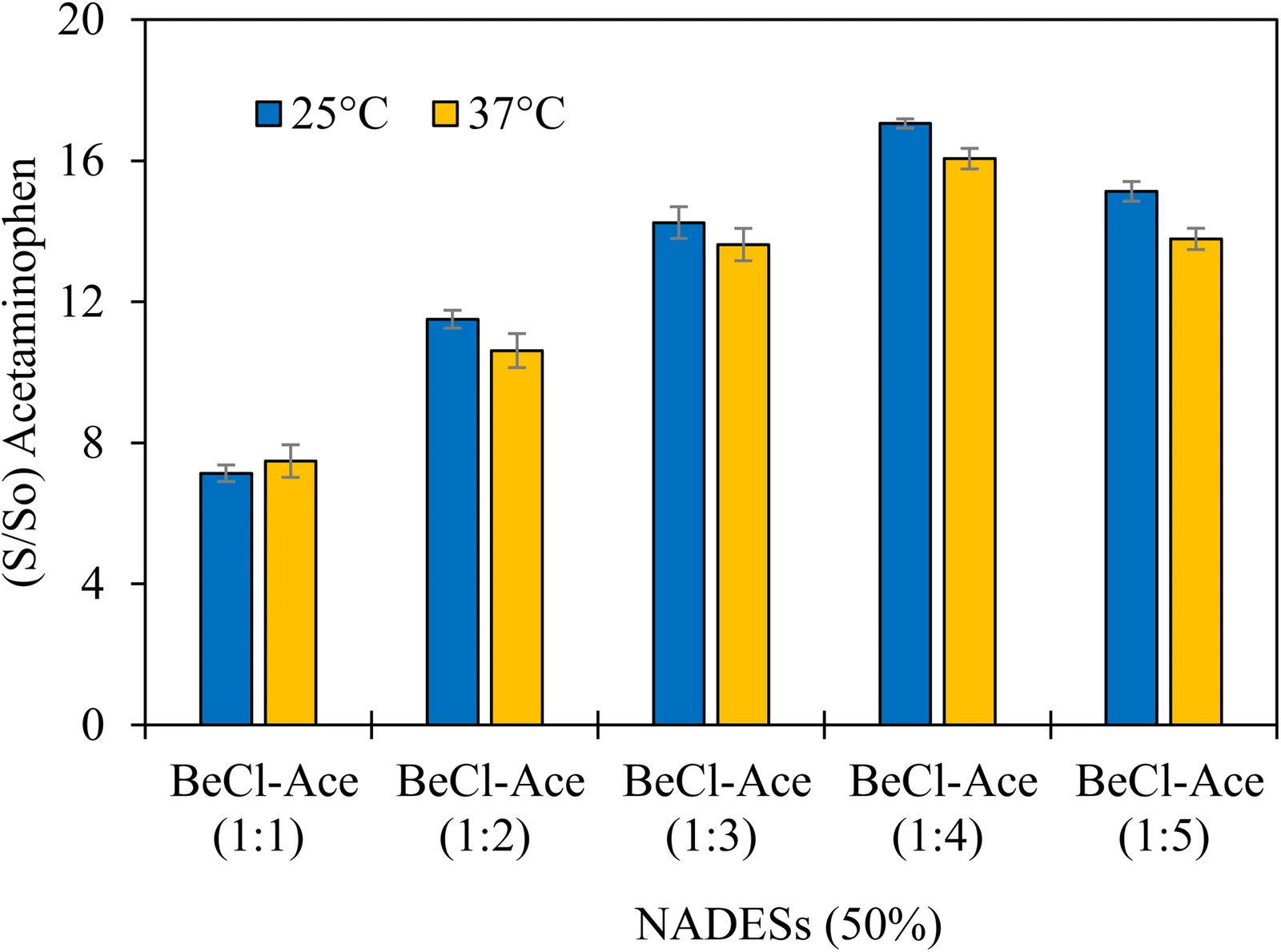
Effect of HBA/HBD molar ratio on the solubility of ACP in aqueous NADESs (50%) based on BeCl–Ace at 25 °C and 37 °C. *S*/*S*_0_ represents the solubility enhancement factor relative to water. BeCl–Ace (1 : 4) achieved the highest *S*/*S*_0_ value, which was significantly greater than those of the other tested molar ratios (*p* < 0.05).

As shown in Fig. 3, the solubility of ACP increased significantly with increasing acetic acid content from a molar ratio of 1 : 1 to 1 : 4. Specifically, the solubility enhancement factor increased from 7.14 to 17.06 at 25 °C and from 7.48 to 16.06 at 37 °C. However, a slight decrease was observed when the ratio was further increased to 1 : 5, indicating that an optimal molar ratio exists for maximizing solubilization efficiency. Consequently, BeCl–Ace (1 : 4) was identified as the optimal formulation.

This trend can be explained by the critical role of HBD content in modulating the hydrogen bonding network within NADESs. Increasing the proportion of acetic acid enhances the availability of hydrogen bond donating sites, thereby facilitating stronger and more extensive intermolecular interactions with ACP molecules. Given that ACP contains both hydroxyl and amide functional groups, it can effectively interact with the HBD through multiple hydrogen bonds, leading to improved disruption of the crystal lattice and enhanced solubilization.

In addition to hydrogen bonding, an increase in HBD content may also modify the polarity and microstructure of the NADES, creating a more favorable solvation environment for ACP. The relatively small molecular size of acetic acid further enables closer molecular packing and more efficient interaction with ACP, contributing to the observed increase in solubility.

However, when the HBD content becomes excessively high, as in the case of the 1 : 5 molar ratio, the solubilization efficiency decreases. This behavior may be attributed to changes in the internal structure of the NADES, where excessive HBD may disrupt the optimal organization of the hydrogen bonding network or increase viscosity, thereby limiting mass transfer and reducing the effective interaction between the solvent and ACP.

Overall, these results demonstrate that the molar ratio of HBD to HBA is a key parameter in determining the solubilization performance of NADESs. An optimal balance between hydrogen bonding capacity, solvent structure, and mass transfer properties is required to achieve maximum solubility. In this study, the BeCl–Ace (1 : 4) system provided the best performance and was therefore selected for subsequent investigations.

#### 3.1.4. Effect of NADES concentration on ACP solubility

Following the optimization of HBD, HBA, and molar ratio, the effect of NADES concentration on ACP solubility was investigated using the selected betaine NADES (BeCl–Ace, 1 : 4). The concentration of aqueous NADESs was systematically varied from 10 to 100% to evaluate the combined influence of solvent composition, hydrogen bonding environment, and viscosity on ACP solubilization. The results are presented in Fig. 4.

**Fig. 4:**
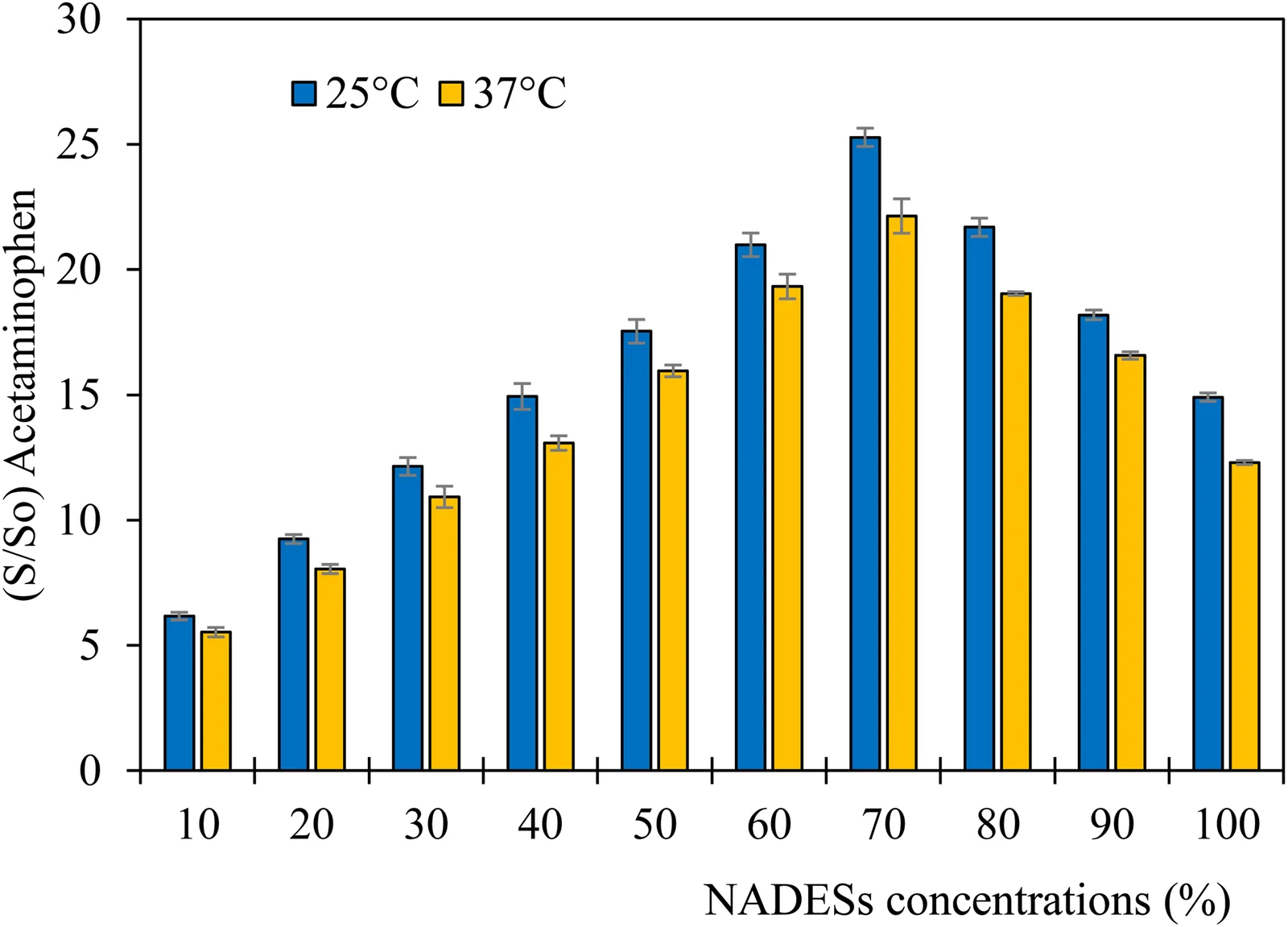
Effect of NADESs concentration on the solubility of ACP in aqueous BeCl–Ace (1 : 4) at 25 °C and 37 °C. *S*/*S*_0_ represents the solubility enhancement factor relative to water. The 70% NADES concentration achieved the highest *S*/*S*_0_ value, which was significantly greater than those of the other tested concentrations (*p* < 0.05).

As shown in Fig. 4, the concentration of NADESs exerted a pronounced effect on ACP solubility. The solubility enhancement factor increased progressively as the NADES concentration increased from 10 to 70%, rising from 6.17 to 25.28 at 25 °C and from 5.52 to 22.14 at 37 °C. However, a different trend was observed beyond 70%. Increasing the NADES concentration to 80%, 90%, and 100% resulted in a gradual decline in solubility, with *S*/*S*_0_ values decreasing to 21.69, 18.19, and 14.91 at 25 °C, and to 19.04, 16.57, and 12.30 at 37 °C, respectively. These findings indicate that an optimal NADES concentration exists for maximizing ACP solubilization, and that excessively high NADES contents do not necessarily provide further improvement.

The effect of NADES concentration on ACP solubilization appears to arise from the competing influence of solvent interaction strength and mass transfer properties. At low NADES concentrations, the system contains a large proportion of water, which weakens the supramolecular hydrogen bonding network between BeCl and Ace. Such dilution reduces the characteristic solvation environment of NADESs and limits favorable interactions with ACP, resulting in relatively low solubility.^33^ Consequently, the solubilization capacity remained limited at 10–30% NADES concentrations.

As the NADES concentration increased, ACP solubility improved markedly, reaching a maximum at 70%. This enhancement can be attributed to the progressively strengthened interaction environment created by the higher abundance of hydrogen bond donors and acceptors. The BeCl–Ace system provides both polar and mildly hydrophobic domains capable of interacting with ACP through hydrogen bonding and dispersion interactions, thereby facilitating crystal lattice disruption and stabilization of the dissolved molecules. At intermediate concentrations, water still plays a beneficial role by maintaining sufficient molecular mobility and preventing excessive viscosity, which favors mass transfer and dissolution.

However, further increasing the NADES concentration beyond 70% resulted in a gradual decline in ACP solubility. Although highly concentrated systems contain abundant interaction sites, their substantially increased viscosity may hinder molecular diffusion and reduce effective solvent–solute contact. In addition, stronger self association and a more rigid internal hydrogen bonding network in concentrated NADESs may reduce the accessibility of interaction sites available for ACP solvation. Therefore, ACP solubilization is governed by a balance between interaction strength and diffusional limitations, with BeCl–Ace (1 : 4) at 70% providing the optimal compromise under the investigated conditions.

A number of studies have investigated the use of DESs/NADESs to enhance the solubility of ACP. DESs based on choline chloride, ethylammonium chloride, tetrapropylammonium bromide, betaine, and choline bitartrate have been reported to significantly improve ACP solubility, with enhancement factors ranging from 4.46 to 17.70 times compared to water.^15^ Dorota *et al.* employed NADESs composed of choline chloride–1,2-propanediol (1 : 2), choline chloride–malic acid (1 : 1), and choline chloride–tartaric acid (1 : 1), demonstrating that ACP solubility strongly depends on NADES concentration, with improvements ranging from 1.13 to 2.63 times.^8^ Similarly, NADESs such as choline chloride–malic acid (1 : 1), choline chloride–malonic acid (1 : 1), and choline chloride–urea (1 : 2) at various concentrations enhanced ACP solubility from 1.67 to 9.79 times.^12^ Other NADES systems, including choline chloride–urea (1 : 2), choline chloride–oxalic acid (1 : 1), and choline chloride–malonic acid (1 : 1), exhibited relatively moderate improvements, with maximum solubility increases of 1.96, 2.07, and 2.17 times, respectively.^14^ Hemayat *et al.* further demonstrated that NADESs based on choline chloride–glycerol (1 : 2) and choline chloride–ethylene glycol (1 : 2) significantly enhanced ACP solubility, with maximum increases of 17.16 and 34.85 times, respectively; however, the inherent toxicity of ethylene glycol poses a major limitation for its pharmaceutical applicability.^13^ In addition, ionic liquids based on choline lactate and choline bitartrate have also been shown to improve ACP solubility, achieving maximum enhancement factors of 2.22–2.65 times at 15% concentration.^7^

In the present study, BeCl–Ace (1 : 4) 70% substantially enhanced ACP solubility, achieving an increase of more than 25-fold. However, direct quantitative comparisons among different DES/NADES systems should be interpreted cautiously because of differences in solvent composition, water content, temperature, and experimental conditions among studies. Importantly, the contribution of the present study extends beyond the magnitude of solubility enhancement by systematically evaluating formulation variables and integrating the optimized BeCl–Ace system with molecular-level interaction analysis, storage stability assessment, and preliminary cytotoxicity evaluation.

#### 3.1.5. Characterization of the selected BeCl–Ace NADES

The selected BeCl–Ace (1 : 4) system was further characterized by FTIR and ^1^H NMR spectroscopy to investigate intermolecular interactions between its components. In the FTIR spectra, the characteristic carbonyl bands of BeCl and Ace at 1725.98 and 1703.84 cm^−1^, respectively, were observed at 1709.46 cm^−1^ in BeCl–Ace, accompanied by changes in the O–H region, indicating modification of the local chemical environments of the functional groups upon mixing (Fig. S1–S4). Consistently, ^1^H NMR analysis showed small but discernible shifts in the characteristic resonances of both components. The BeCl–CH_2_– and N^+^(CH_3_)_3_ signals shifted from 4.405 and 3.264 ppm to approximately 4.38 and 3.248 ppm, respectively, while the Ace methyl signal shifted from approximately 1.92 to 1.90 ppm. In addition, the distinct carboxylic proton signal of Ace at 11.908 ppm was markedly altered in the BeCl–Ace system (Fig. S5–S8). Collectively, these FTIR and ^1^H NMR spectral changes indicate a reorganization of the local chemical environments of BeCl and Ace upon mixing and are consistent with intermolecular association, particularly hydrogen-bonding interactions, thereby providing experimental support for the formation of the BeCl–Ace NADES.

### 3.2. Molecular mechanism of acetaminophen solubilization in NADES

#### 3.2.1. ESP analysis

The ESP analysis and binding energy calculations indicate that ACP interacts more favorably with the components of the BeCl–Ace green solvent system than with water, providing a molecular rationale for the experimentally observed solubility enhancement. As shown in Fig. 5A, the ESP surface of ACP exhibits electron-rich regions mainly localized around the oxygen atoms of the phenolic hydroxyl and amide carbonyl groups, together with electron-deficient regions associated with hydrogen atoms capable of acting as hydrogen-bond donors. This charge distribution creates favorable conditions for polar interactions and hydrogen bonding with the solvent components.

**Fig. 5:**
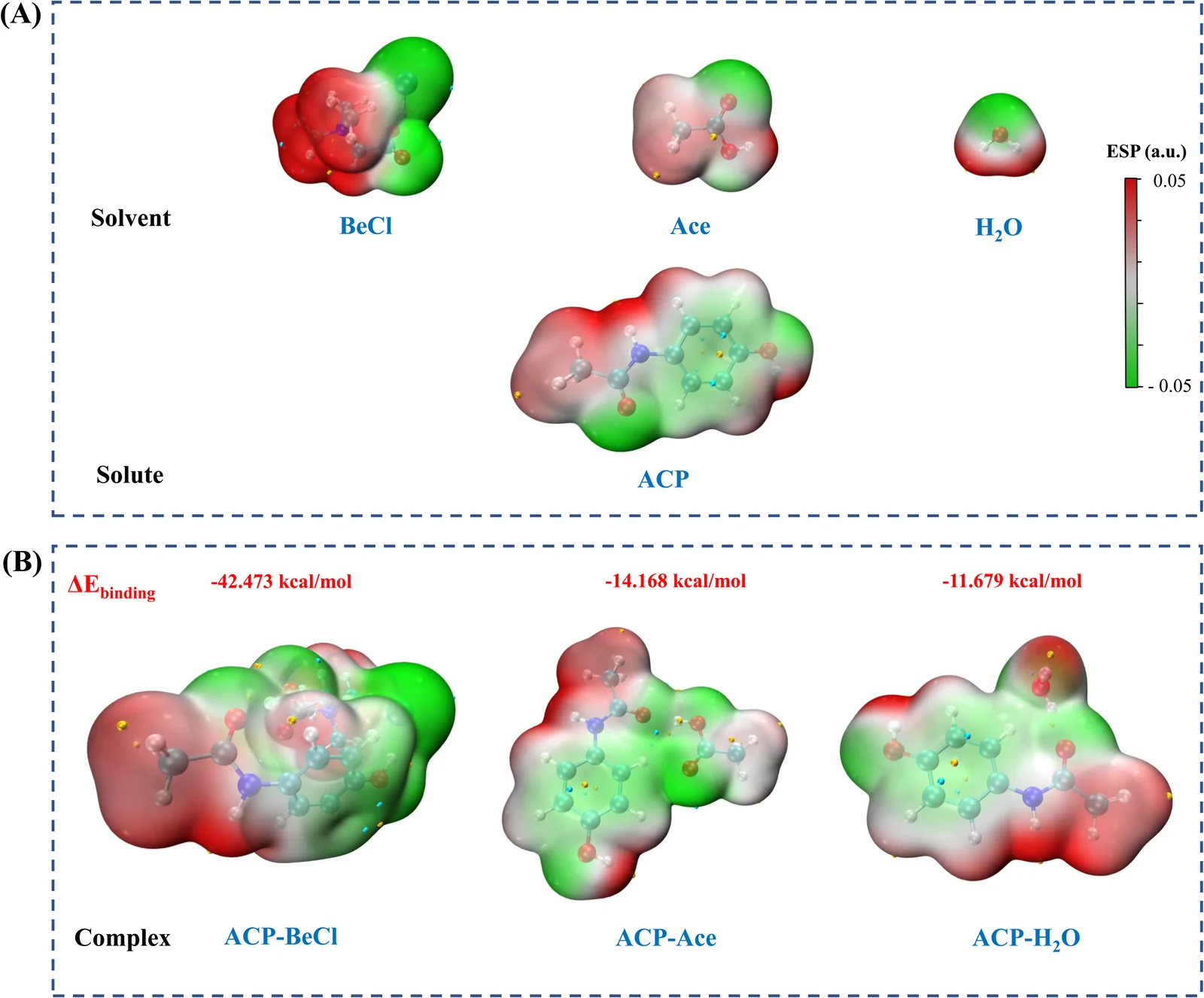
ESP maps and binding energies of ACP and its complexes with solvent components. (A) ESP surfaces of BeCl, Ace, H_2_O, and ACP (red: electron-deficient; green: electron-rich). (B) Optimized structures and binding energies (Δ*E*_binding_) of ACP–BeCl, ACP–Ace, and ACP–H_2_O complexes.

Quantitatively, the binding energies follow the order ACP–BeCl (−42.473 kcal mol^−1^) ≫ ACP–Ace (−14.168 kcal mol^−1^) > ACP–H_2_O (−11.679 kcal mol^−1^) (Fig. 5B), showing that ACP has a markedly stronger molecular affinity for the components of the BeCl–Ace system than for water alone, particularly for BeCl. These results suggest that ACP is preferentially stabilized by specific interactions with BeCl and Ace rather than by the weaker interactions present in water. In this context, BeCl plays the dominant role in stabilizing ACP, while Ace contributes by further enhancing the interaction environment. Therefore, the improved solubility of ACP in the BeCl–Ace system can be rationalized by the formation of a more favorable solvation environment than that provided by water, which more effectively stabilizes the dissolved state of the drug.

#### 3.2.2. IRI analysis

The IRI analysis provides further insight into the nature and relative strength of the noncovalent interactions between ACP and BeCl, Ace, and H_2_O, thereby supporting the enhanced solubility observed in the BeCl–Ace system. The scatter plots of sign(*λ*_2_)*ρ versus* IRI show that most data points for all three complexes are located in the negative or near-zero sign(*λ*_2_)*ρ* region, indicating that attractive interactions dominate, mainly in the form of hydrogen bonding and van der Waals interactions. Nevertheless, clear differences are observed among the three complexes.

For the ACP–BeCl complex, the data points extend more broadly into the negative sign(*λ*_2_)*ρ* region and form a dense distribution from moderately negative values toward near-zero values (Fig. 6A), reflecting the coexistence of strong directional interactions and extensive dispersion contacts. In comparison, the ACP–Ace scatter plot is narrower and more concentrated near the zero region (Fig. 6B), indicating that although attractive interactions are present, their extent and diversity are lower than in ACP–BeCl. The ACP–H_2_O complex exhibits the most localized distribution, with a relatively narrow negative region (Fig. 6C), suggesting that the interaction between ACP and water is mainly restricted to a localized attractive contact rather than an extended stabilization region.

**Fig. 6:**
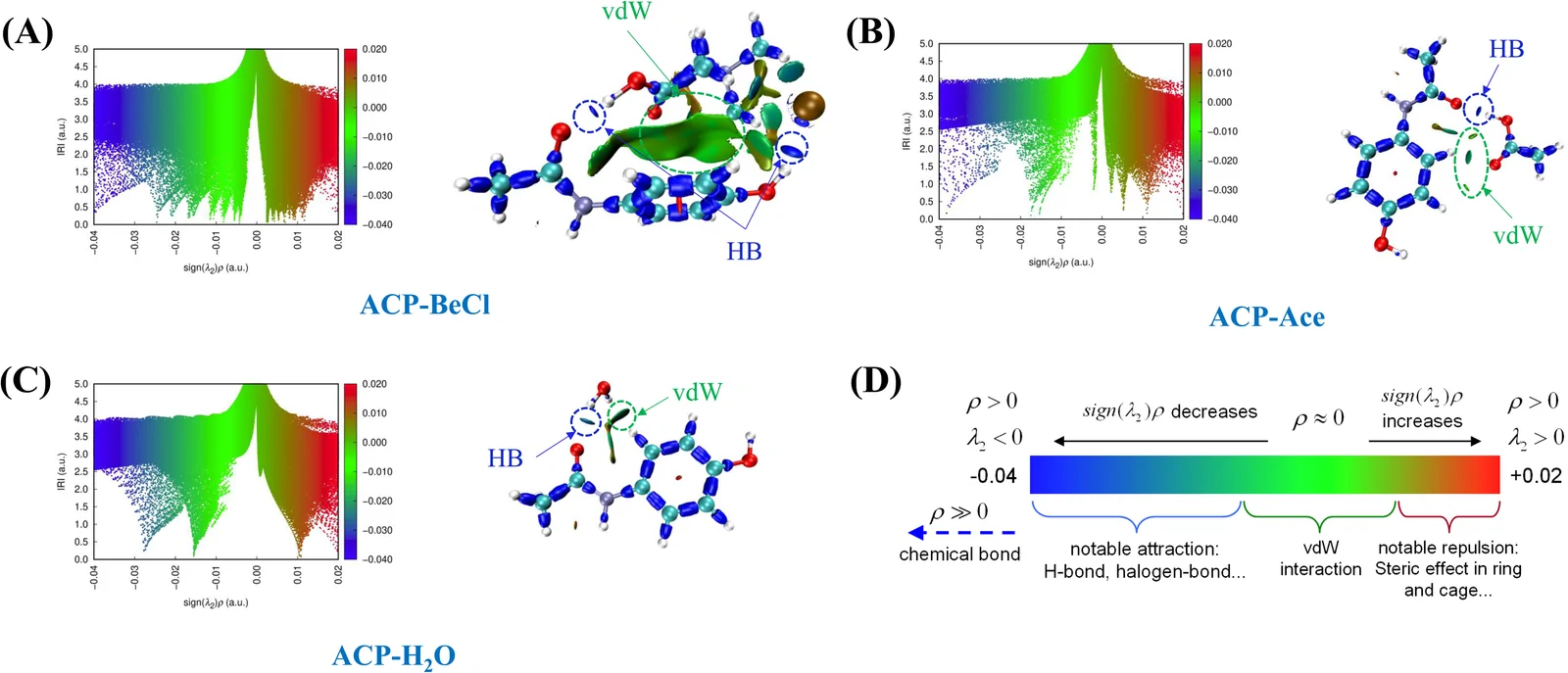
IRI analysis of ACP–solvent complexes. (A–C) Scatter plots of sign(*λ*_2_)*ρ versus* IRI and corresponding IRI isosurfaces for ACP–BeCl, ACP–Ace, and ACP–H_2_O complexes. (D) Color scale used for identifying noncovalent interactions (blue: strong attraction; green: van der Waals interaction; red: repulsion).

These trends are consistent with the IRI isosurface analysis. The ACP–BeCl complex shows multiple blue regions corresponding to hydrogen-bonding interactions, together with large and continuous green regions representing van der Waals contacts over a broad interfacial area. This indicates that ACP–BeCl is stabilized not only by localized directional interactions but also by extensive dispersion interactions across a wide contact surface, in agreement with its most negative binding energy. In contrast, the ACP–Ace complex also exhibits both hydrogen-bonding and van der Waals interactions, but these regions are more localized and less continuous, indicating weaker overall stabilization than in ACP–BeCl. For ACP–H_2_O, the isosurface is characterized mainly by a single hydrogen bond accompanied by a small and discontinuous van der Waals region, reflecting a much simpler interaction pattern. Thus, while water can form an effective localized attractive interaction with ACP, it does not create the extended and cooperative noncovalent interaction environment observed with the green solvent components.

Taken together, the scatter plot and isosurface analyses show that the enhanced solubility of ACP in the BeCl–Ace system is not determined solely by the presence of hydrogen bonding, but also by the extent, continuity, and cooperativity of the noncovalent interaction regions between ACP and the solvent components. BeCl makes the greatest contribution by providing both strong directional interactions and extensive van der Waals contacts, thereby stabilizing ACP more effectively than water. Although Ace contributes to a lesser extent, it still enhances the interaction environment through additional attractive and dispersive contacts. As a result, the BeCl–Ace system provides a solvation environment richer in stabilizing interactions than water, which is expected to improve the stabilization of ACP in the dissolved state and thereby increase its apparent solubility.

From a formulation perspective, the release behavior of ACP from BeCl–Ace may also be influenced by diffusional transport and the composition of the receiving medium. The structured intermolecular environment and relatively high viscosity of concentrated NADESs could restrict molecular mobility and consequently influence ACP diffusion.^34,35^ Upon contact with an aqueous medium, progressive dilution may weaken intermolecular interactions within the NADES and alter its physicochemical and solvation properties, potentially affecting ACP transport and release.^36,37^ The effect of pH is expected to be relatively limited within the usual physiological range because ACP is a weak acid (p*K*_a_ ≈ 9.5) and remains predominantly unionized below this pH; however, under sufficiently alkaline conditions approaching or exceeding its p*K*_a_, increased ionization could enhance its apparent solubility and thereby modify its release behavior.^38,39^ Because the present study focused on equilibrium solubility rather than release kinetics, these effects were not experimentally evaluated. Dedicated release studies considering both medium composition and pH are therefore required to establish the release mechanism and kinetics of ACP from BeCl–Ace-based formulations.

### 3.3. Stability of ACP in water and NADESs

The stability of ACP in pure water and in the selected NADES formulation, BeCl–Ace (1 : 4) 70%, was evaluated after 15 and 30 days of storage at 25 °C and 37 °C. The results are presented in Fig. 7.

**Fig. 7:**
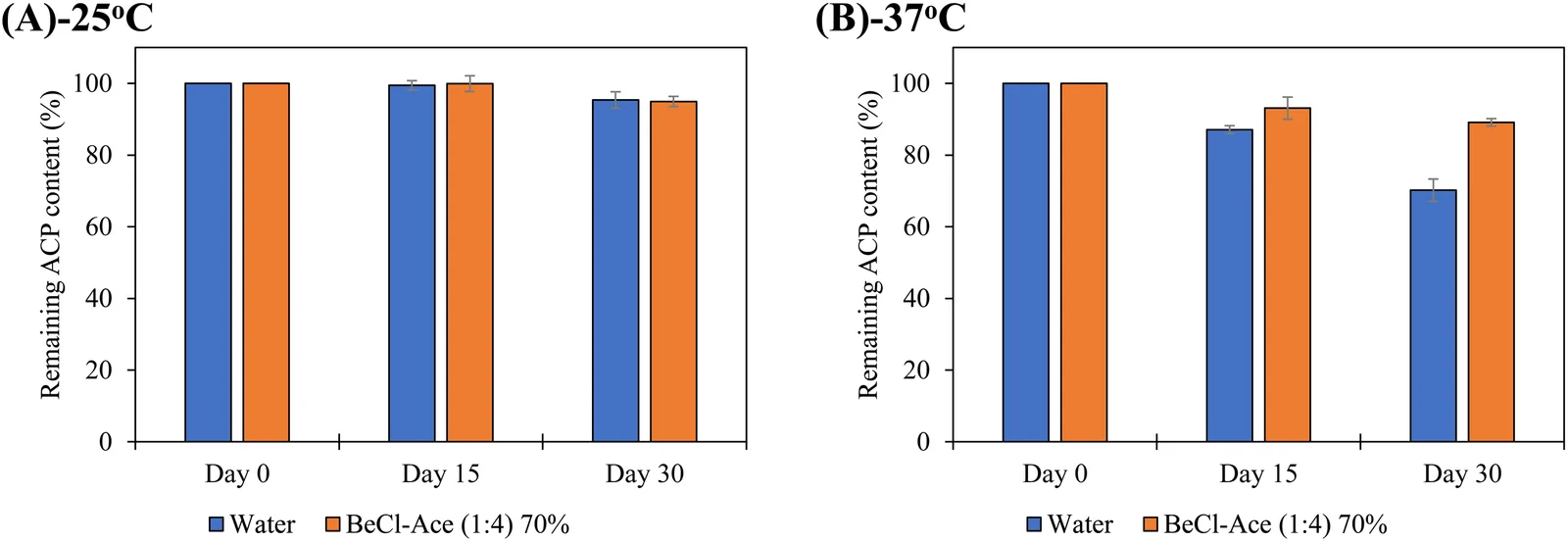
Stability of ACP in pure water and BeCl–Ace (1 : 4) 70% after 15 and 30 days of storage at 25 °C (A) and 37 °C (B). Data are expressed as remaining ACP content (%).

At 25 °C, ACP remained relatively stable in both media over the investigated period. After 15 days of storage, the remaining ACP content was 99.44% in water and 99.92% in BeCl–Ace (1 : 4) 70%. After 30 days, the corresponding values decreased slightly to 95.37% and 94.94%, respectively. These results indicate that ACP exhibited good stability at ambient temperature, and no substantial difference was observed between water and the NADES formulation under these conditions.

In contrast, a more pronounced difference was observed at 37 °C. After 15 days, the remaining ACP content in water decreased to 87.09%, whereas that in BeCl–Ace (1 : 4) 70% remained at 93.09%. After 30 days of storage, ACP retained only 70.20% of its initial content in water, while 89.13% remained in the NADES formulation. These findings clearly demonstrate that BeCl–Ace (1 : 4) 70% significantly improved the stability of ACP under elevated temperature conditions.

The higher apparent ACP retention observed in the NADES medium may be associated with the intermolecular environment provided by the BeCl–Ace system. The hydrogen-bonding interactions suggested by the molecular-level analyses could influence the local solvation environment and molecular mobility of ACP. However, the UV-Vis method used in the present stability study does not specifically distinguish intact ACP from potential degradation products; therefore, specific degradation products and degradation pathways could not be identified. Consequently, the observed differences should be interpreted as differences in apparent ACP retention rather than definitive evidence that BeCl–Ace suppresses a particular degradation pathway. Further studies using validated stability-indicating chromatographic methods are required to confirm this effect and elucidate the underlying degradation mechanisms.

Overall, the results indicate that the stabilizing effect of the selected NADES formulation becomes particularly evident under thermal stress, while at 25 °C both water and BeCl–Ace (1 : 4) 70% maintained ACP at high levels over 30 days. Therefore, in addition to its superior solubilization performance, BeCl–Ace (1 : 4) 70% also provided improved storage stability for ACP, highlighting its potential as a promising solvent system for pharmaceutical formulation.

### 3.4. Cytotoxicity evaluation of ACP loaded NADESs on HEK-293A cells

The cytotoxicity of ACP, NADESs, and ACP loaded NADESs was evaluated on normal human embryonic kidney cells (HEK-293A) using the SRB assay. The tested ACP concentrations ranged from 0.05 to 0.75 µg mL^−1^, and the results are presented in Fig. 8.

**Fig. 8:**
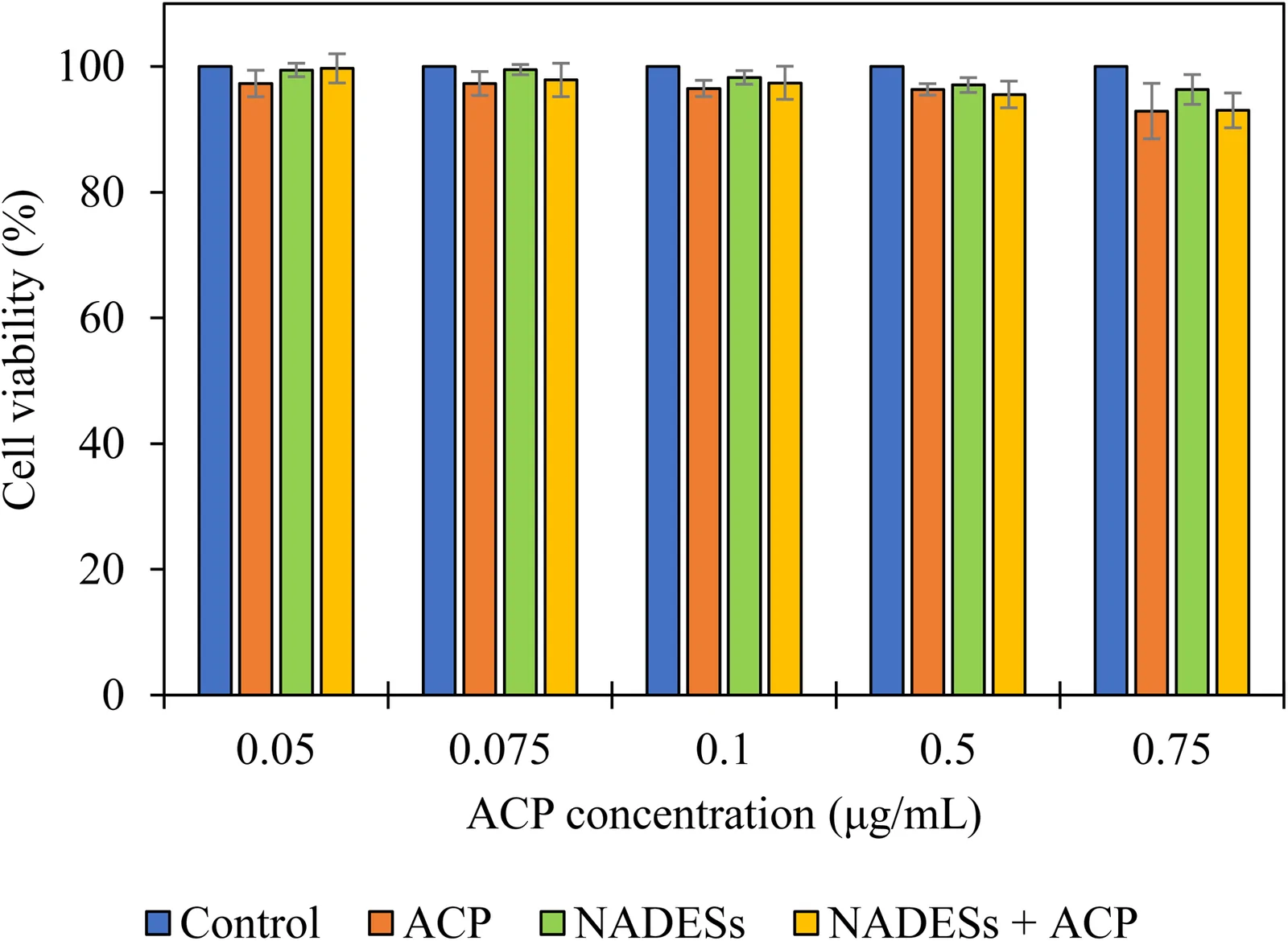
Cell viability of HEK-293A cells treated with ACP, NADESs, and ACP loaded NADESs at different ACP concentrations (0.05–0.75 µg mL^−1^), determined by the SRB assay after 72 h exposure.

As shown in Fig. 8, all tested samples maintained high cell viability across the investigated concentration range. ACP alone exhibited low cytotoxicity toward HEK-293A cells, with cell viability remaining above 92% even at the highest concentration of 0.75 µg mL^−1^. Similarly, NADESs alone, tested at the corresponding final concentrations of 0.099–1.491 µg mL^−1^, maintained cell viability between 96.36% and 99.50%, indicating low cytotoxicity under the tested exposure conditions.

The ACP loaded NADESs formulation also maintained high cell viability, with values ranging from 93.02 to 99.70%. Although a slight concentration dependent decrease in viability was observed, the viability remained above 92% at all tested concentrations, suggesting that the incorporation of ACP into NADESs did not increase cytotoxicity toward HEK-293A cells. These results indicate that the selected NADESs formulation is biocompatible and suitable for further investigation.

The morphological observations further supported the quantitative results. As shown in Fig. 9, HEK-293A cells treated with ACP, NADESs, and ACP loaded NADESs retained normal morphology, with no obvious signs of severe cell shrinkage, detachment, or significant loss of cell density across the investigated concentrations. At higher concentrations, only slight reductions in cell density were observed, which were consistent with the minor decrease in cell viability obtained from the SRB assay.

**Fig. 9:**
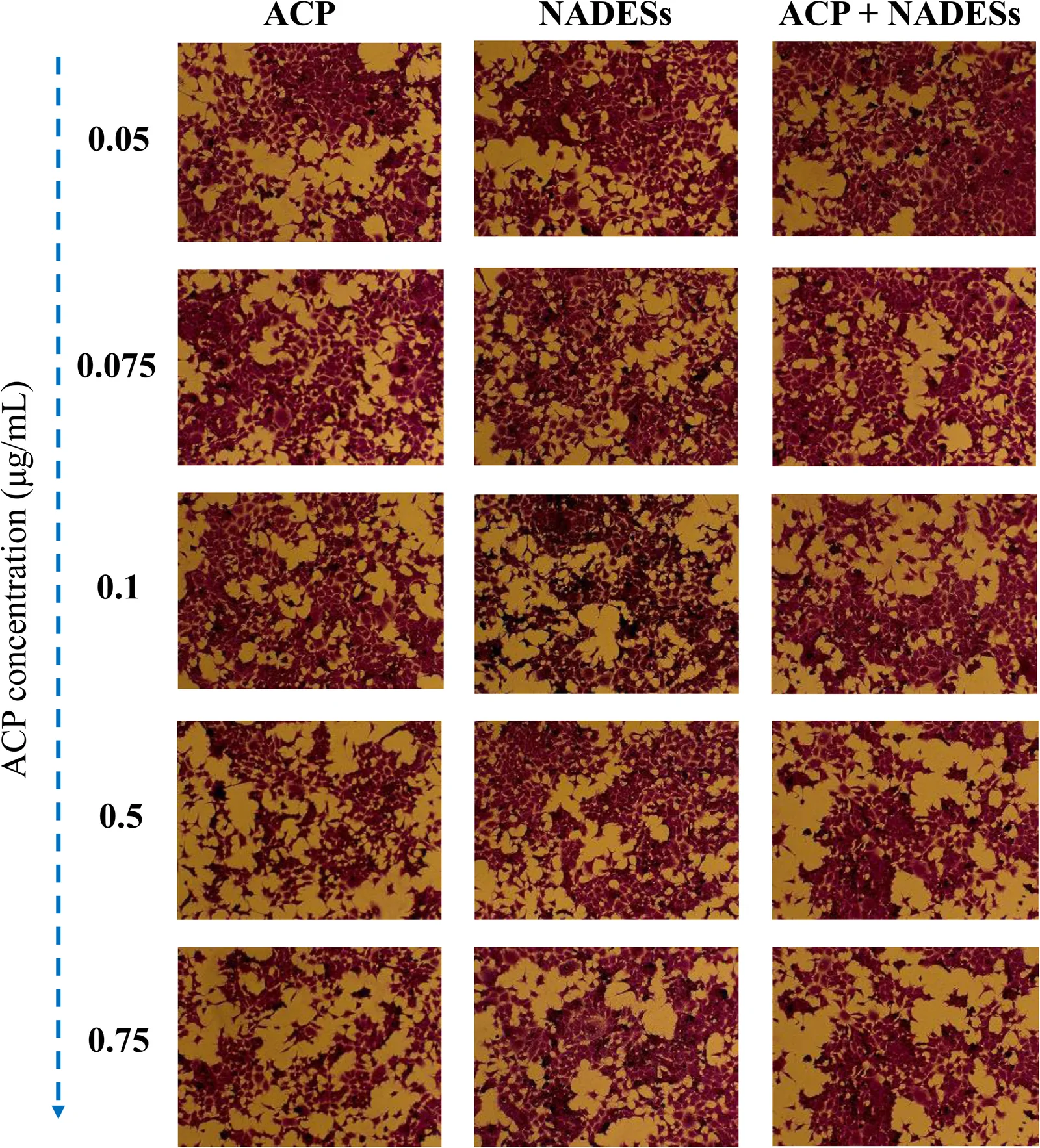
Representative microscopic images of HEK-293A cells treated with ACP, NADESs, and ACP loaded NADESs at different ACP concentrations (0.05–0.75 µg mL^−1^) after 72 h exposure.

Overall, the results demonstrate that the selected NADESs formulation exhibited low cytotoxicity toward normal HEK-293A cells and did not potentiate the cytotoxic effect of ACP. This finding, together with the enhanced solubility and stability observed in previous sections, highlights the potential of NADESs as a promising solvent system for ACP formulation. However, the BeCl–Ace concentrations evaluated in the present cytotoxicity assay represent the final concentrations after dilution of the ACP-loaded formulation into the cell culture medium and therefore do not directly assess the cytocompatibility of the concentrated 70% aqueous NADES formulation. Further studies over a broader range of defined BeCl–Ace concentrations are required to establish concentration-dependent cytocompatibility and further evaluate its applicability in pharmaceutical systems.

## 4. Conclusion

This study demonstrates that aqueous NADESs are highly effective green solvent systems for improving the solubility and formulation performance of acetaminophen. Systematic screening revealed that the solubilization behavior of ACP strongly depends on the chemical nature of both HBD and HBA, their molar ratio, and the NADES concentration. Among the investigated systems, BeCl–Ace was identified as the optimal formulation, with BeCl–Ace (1 : 4) at 70% providing the highest solubilization efficiency, increasing ACP solubility by more than 25-fold relative to water. Computational analyses provided molecular level insights into the enhanced solubilization behavior. ESP mapping, binding energy calculations, and IRI analysis consistently showed that ACP interacts much more favorably with BeCl and Ace than with water, owing to a cooperative interaction network involving strong hydrogen bonding, electrostatic attraction, and extensive van der Waals stabilization. In particular, BeCl plays a dominant role in molecular recognition and stabilization of ACP, while Ace further strengthens the overall solvation environment, together creating a structured solvent network capable of efficiently stabilizing dissolved ACP molecules. Beyond solubility enhancement, the optimized NADES formulation significantly improved the storage stability of ACP, especially under elevated temperature conditions, and maintained excellent biocompatibility toward normal HEK-293A cells, with cell viability consistently exceeding 92%. The combination of high solubilization capacity, improved stability, low cytotoxicity, and clear mechanistic understanding highlights the strong potential of aqueous BeCl–Ace NADESs as sustainable pharmaceutical excipients. Overall, this work not only establishes an efficient green solvent platform for ACP formulation, but also provides a rational mechanistic framework for designing NADES-based solvent systems to improve the performance of other poorly or moderately water soluble pharmaceutical compounds.

## Author contributions

The-Huan Tran: methodology, investigation, software, writing – original draft preparation; Thao Nhu Thi Nguyen: investigation, formal analysis; Quynh Phuong Nguyen: investigation, formal analysis; Nhan Trong Le: conceptualization, methodology, writing – original draft preparation, supervision; Hoai Thi Nguyen: conceptualization, methodology, writing – review and editing, supervision.

## Conflicts of interest

There are no conflicts to declare.

## Supplementary Material

RA-016-D6RA05672E-s001

## Data Availability

Data will be made available on request. Supplementary information (SI) is available. See DOI: https://doi.org/10.1039/d6ra05672e.
