# Supplementary material for: Enhancing acetaminophen solubility and stability using an aqueous betaine chloride–acetic acid natural deep eutectic solvent: experimental and mechanistic insights
Source: RSC Adv. 2026 Oct 12;16(47):62140–52. doi: 10.1039/d6ra05672e (PMC13655794; doi:10.1039/d6ra05672e)
Supplement: Supplementary file 1 [file RA-016-D6RA05672E-s001.pdf]

Supplementary Information (SI) for RSC Advances.
This journal is © The Royal Society of Chemistry

Supplementary material

Enhancing acetaminophen solubility and stability using an aqueous betaine chloride–acetic acid natural deep eutectic solvent: Experimental and mechanistic insights

The-Huan Tran,[a] Thao Nhu Thi Nguyen,[a] Quynh Phuong Nguyen,[a] Nhan Trong Le,*[a] and Hoai Thi Nguyen*[a]

[a]Faculty of Pharmacy, Hue University of Medicine and Pharmacy, Hue University, Hue City, Vietnam

* Correspondence: Hoai Thi Nguyen, e-mail: nthoai@hueuni.edu.vn; and Nhan Trong Le, e-mail: ltrongnhan@hueuni.edu.vn; Faculty of Pharmacy, Hue University of Medicine and Pharmacy, Hue University, Hue City, Vietnam.

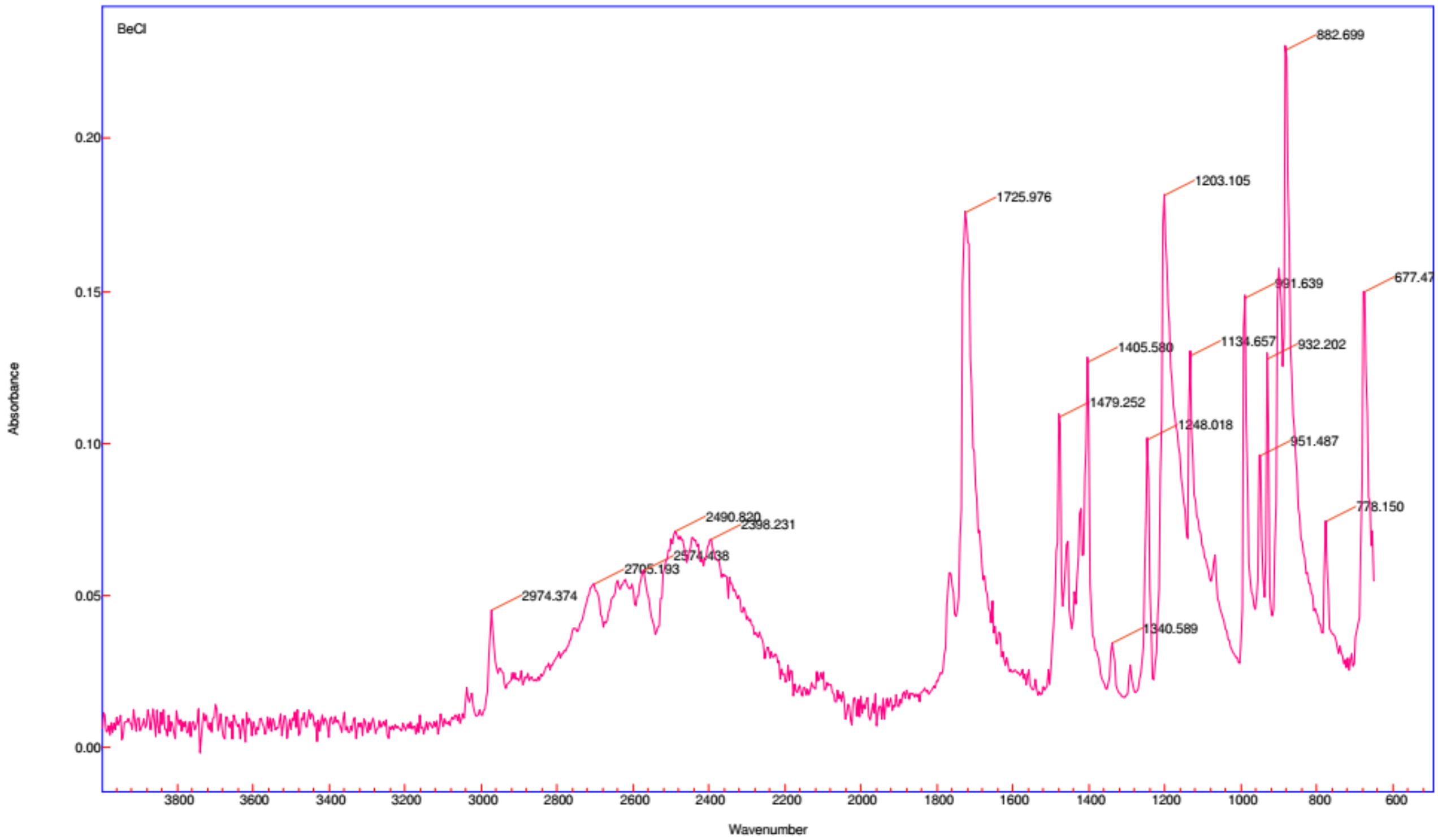

Figure S1. FTIR spectrum of betaine chloride (BeCl).

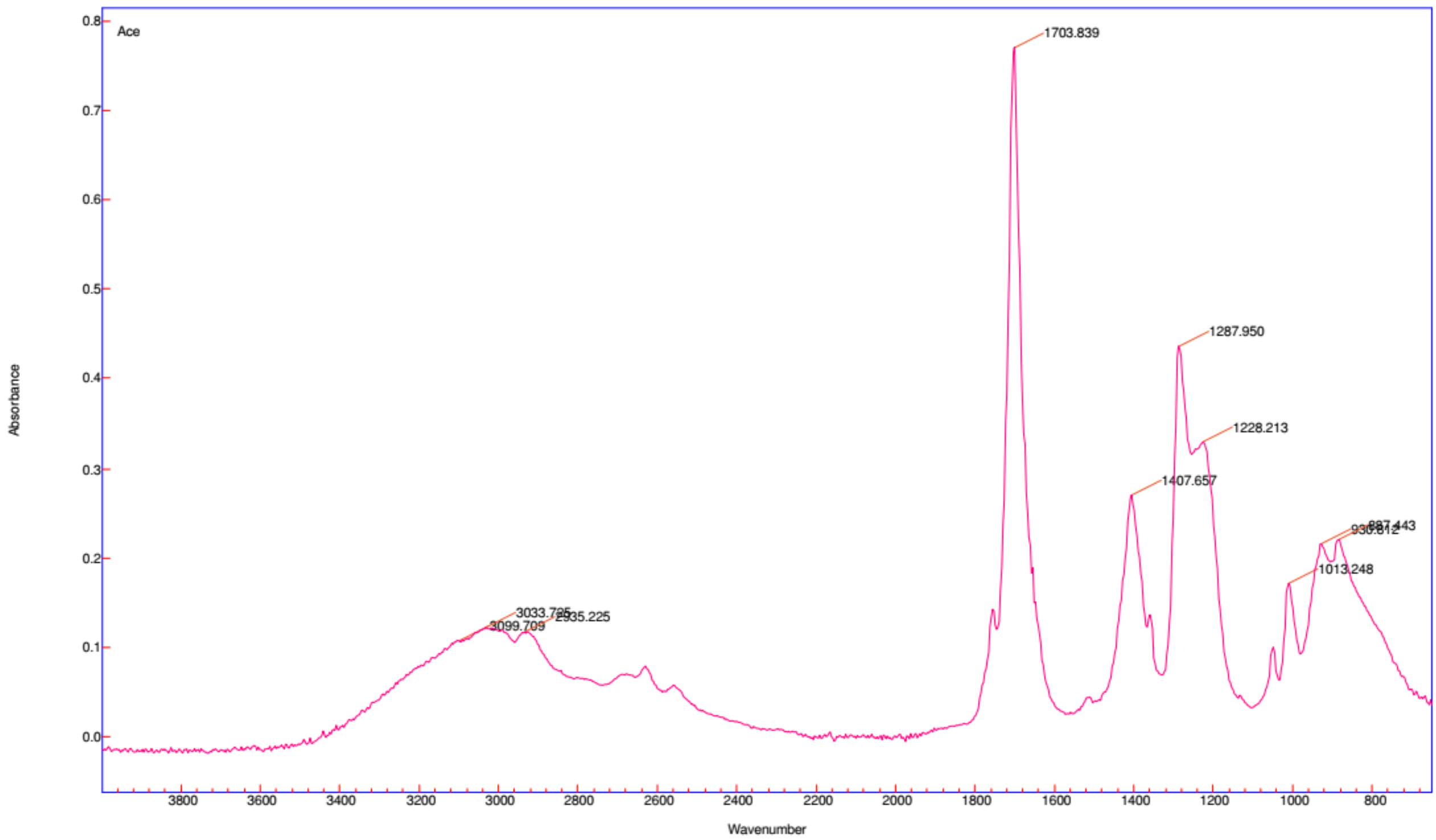

Figure S2. FTIR spectrum of acetic acid (Ace).

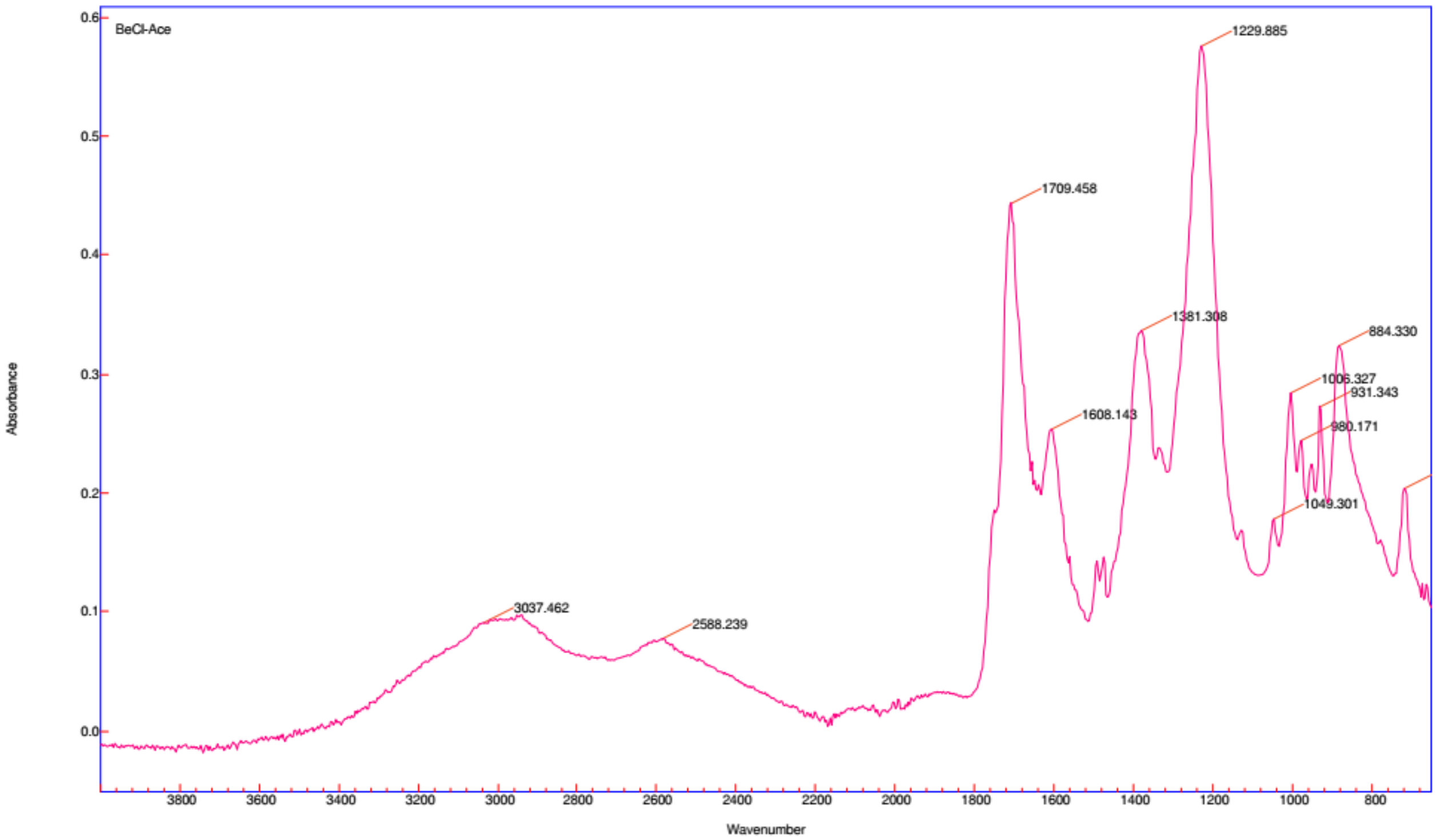

Figure S3. FTIR spectrum of BeCl–Ace.

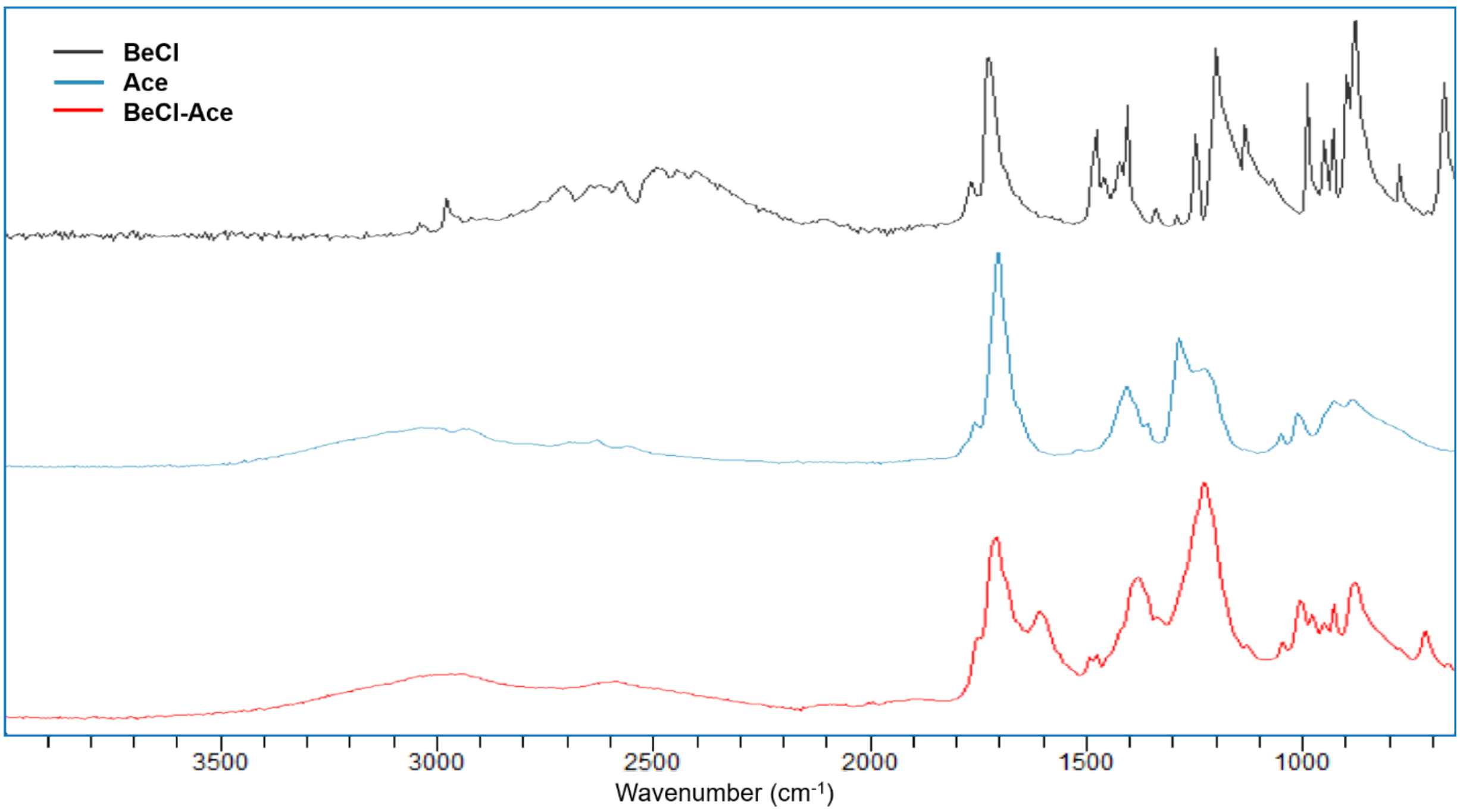

Figure S4. Overlaid FTIR spectra of BeCl, Ace, and BeCl–Ace.

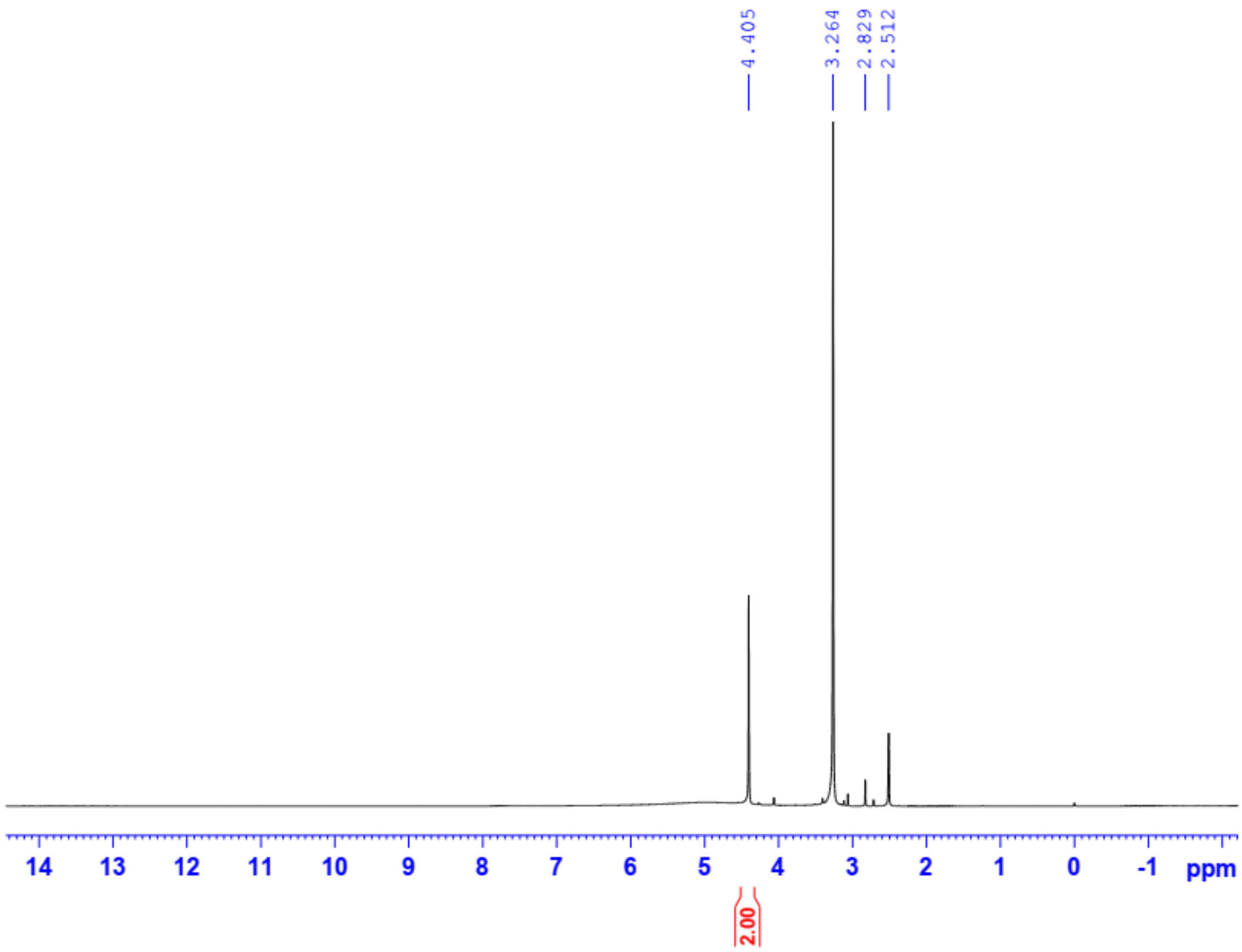

Figure S5. ^{1}H NMR spectrum of BeCl.

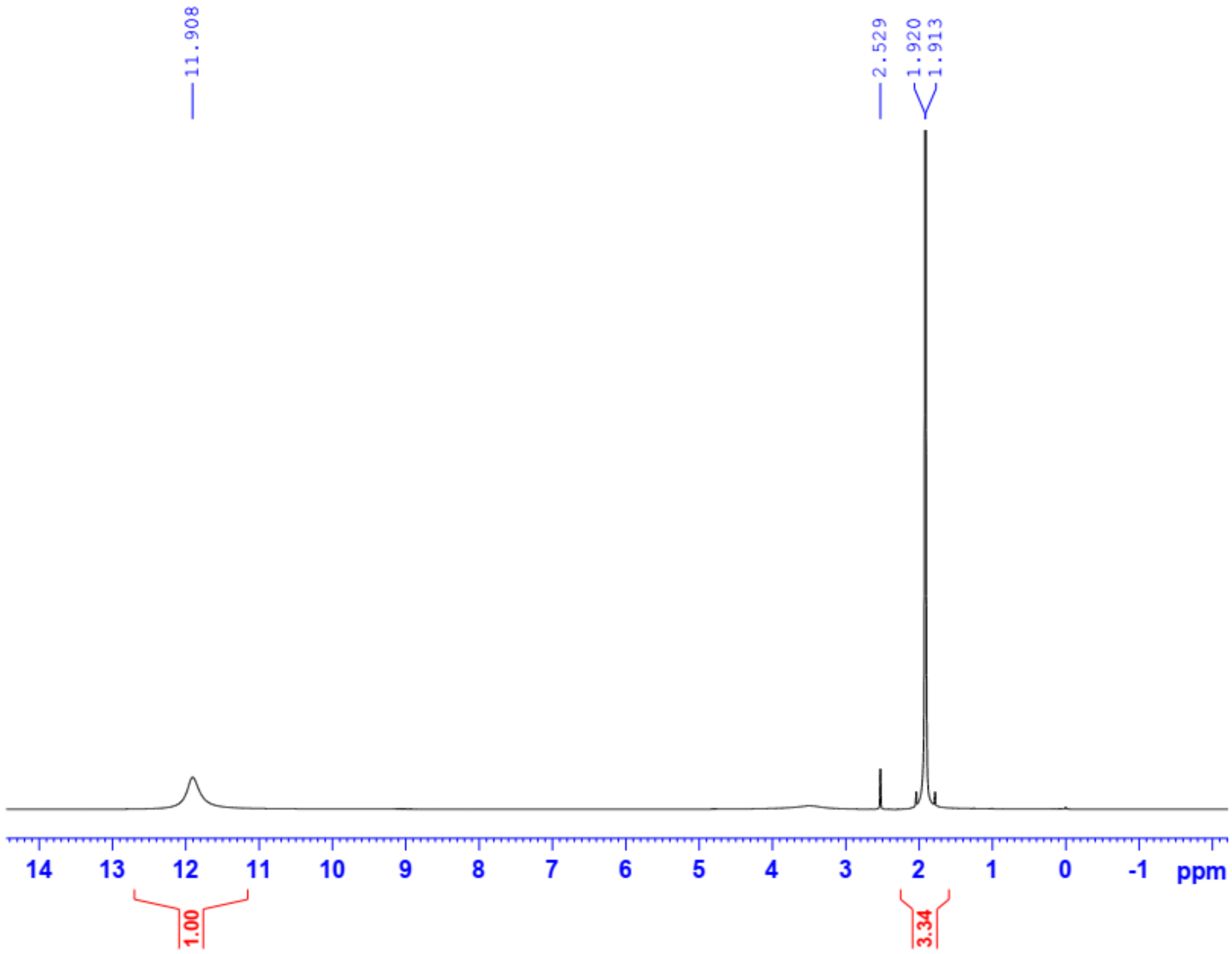

Figure S6. ^{1}H NMR spectrum of Ace.

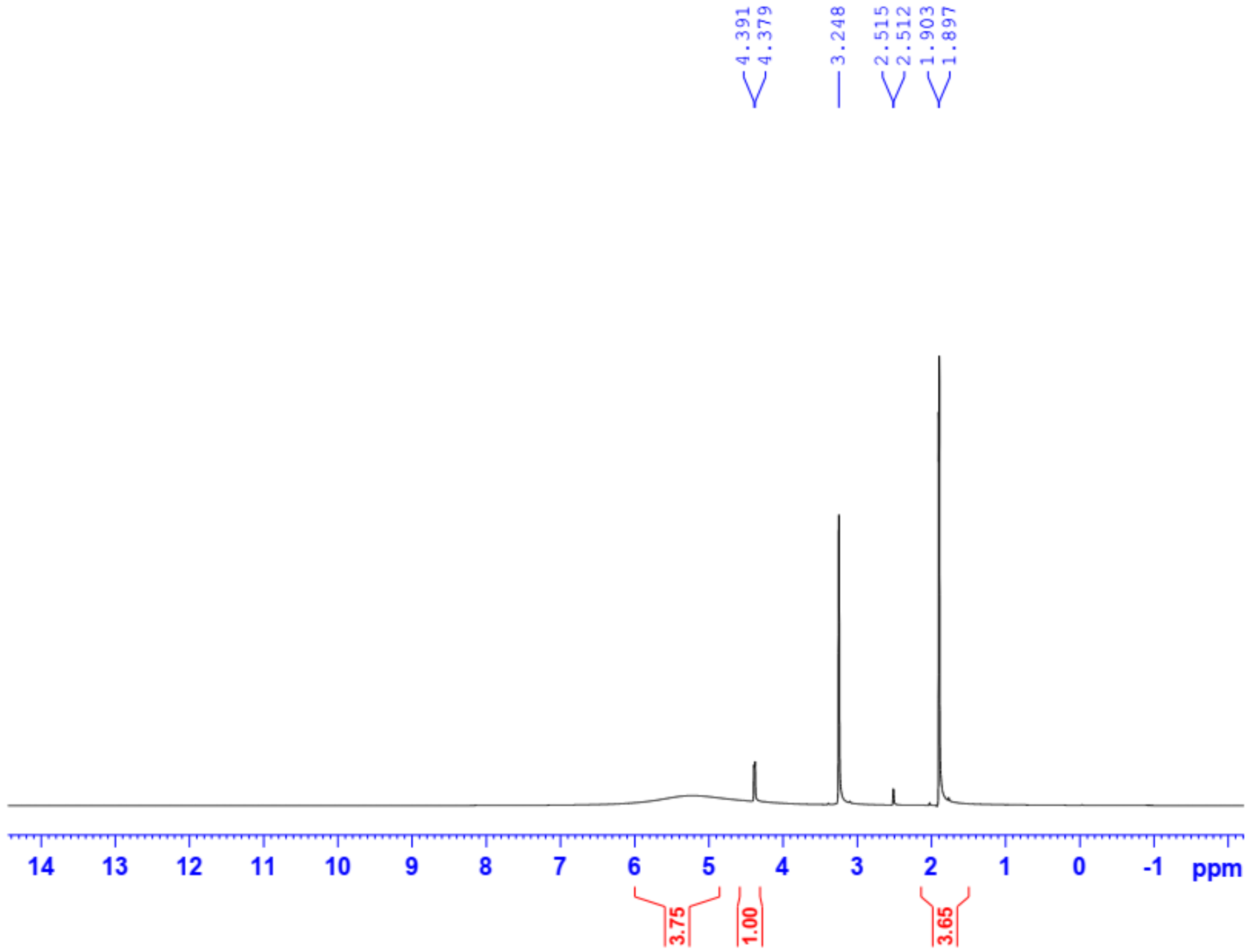

Figure S7. ^{1}H NMR spectrum of BeCl-Ace

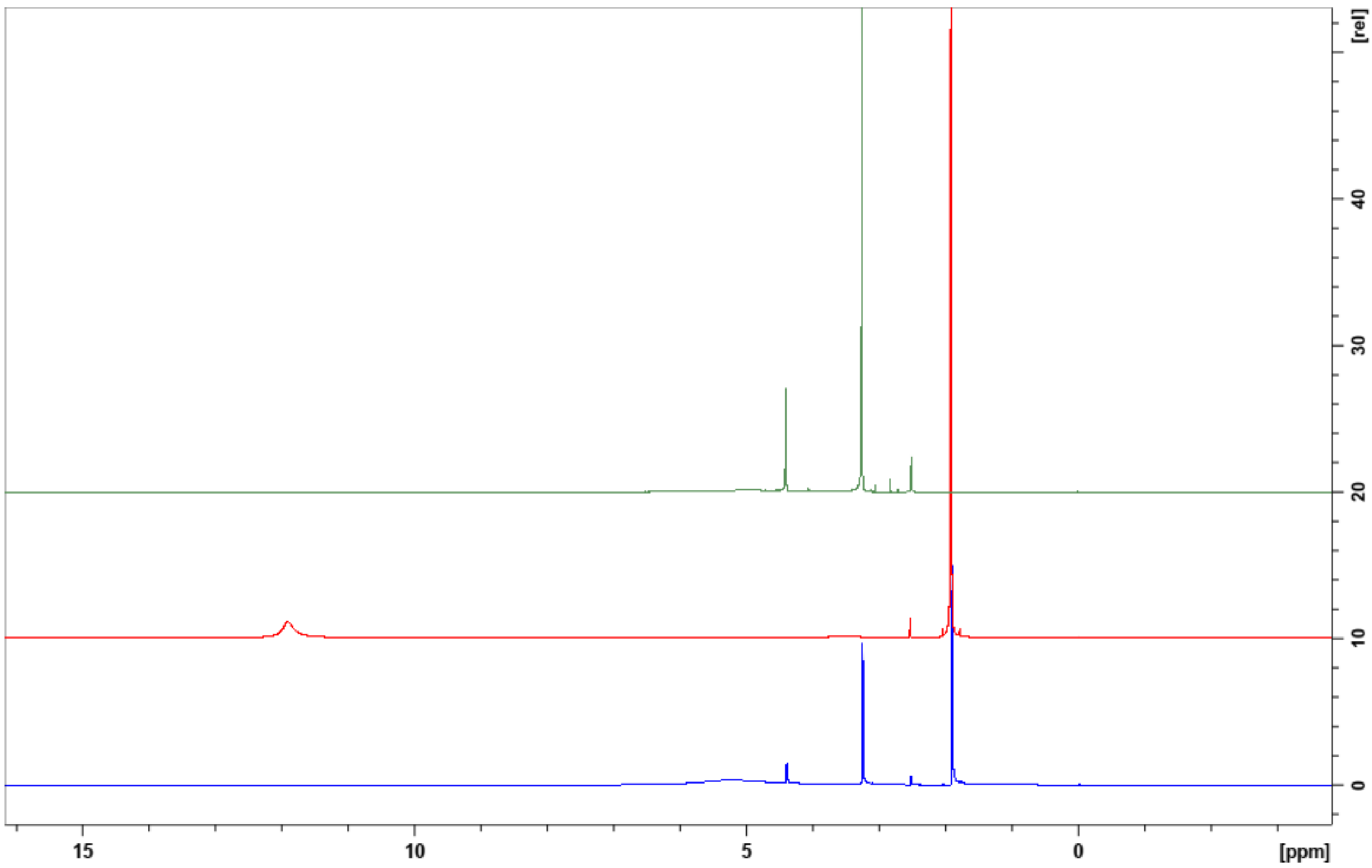

Figure S8. Overlaid ^{1}H NMR spectra of BeCl, Ace, and BeCl–Ace.
